# Predicting climate heating impacts on riverine fish species diversity in a biodiversity hotspot region

**DOI:** 10.1038/s41598-023-41406-9

**Published:** 2023-09-01

**Authors:** Toktam Makki, Hossein Mostafavi, Ali Akbar Matkan, Roozbeh Valavi, Robert M. Hughes, Shabnam Shadloo, Hossein Aghighi, Asghar Abdoli, Azad Teimori, Soheil Eagderi, Brian W. Coad

**Affiliations:** 1 Department of Biodiversity and Ecosystem Management, Environmental Sciences Research Institute, Shahid Beheshti University, Tehran, Iran; 2 The Center for Remote Sensing and Geographic Information System Research, Faculty of Earth Sciences, Shahid Beheshti University, Tehran, Iran; 3 CSIRO Environment, Clayton South, VIC 3169 Australia; 4 Amnis Opes Institute, Corvallis, OR 97333 USA; 5 Department of Fisheries, Wildlife, and Conservation Sciences, Oregon State University, Corvallis, OR 97331 USA; 6 Institute for Oceans and Fisheries, University of British Columbia, Vancouver, Canada; 7 Department of Biology, Faculty of Sciences, Shahid Bahonar University of Kerman, Kerman, Iran; 8 Department of Fisheries, Faculty of Natural Resources, University of Tehran, Karaj, Iran; 9 Canadian Museum of Nature, Ottawa, ON K1P 6P4 Canada

**Keywords:** Ecology, Zoology

## Abstract

Co-occurring biodiversity and global heating crises are systemic threats to life on Earth as we know it, especially in relatively rare freshwater ecosystems, such as in Iran. Future changes in the spatial distribution and richness of 131 riverine fish species were investigated at 1481 sites in Iran under optimistic and pessimistic climate heating scenarios for the 2050s and 2080s. We used maximum entropy modeling to predict species’ potential distributions by hydrologic unit (HU) occupancy under current and future climate conditions through the use of nine environmental predictor variables. The most important variable determining fish occupancy was HU location, followed by elevation, climate variables, and slope. Thirty-seven species were predicted to decrease their potential habitat occupancy in all future scenarios. The southern Caspian HU faces the highest future species reductions followed by the western Zagros and northwestern Iran. These results can be used by managers to plan conservational strategies to ease the dispersal of species, especially those that are at the greatest risk of extinction or invasion and that are in rivers fragmented by dams.

## Introduction

Freshwaters, especially riverine ecosystems are severely affected by climate heating because they are relatively rare compared to terrestrial ecosystems and their thermal and hydrological features are highly dependent on climate. Other pressures, including non-native organisms, pollution, land-use change, over-exploitation, habitat loss, and dams, make them even more vulnerable to climate heating^1^. Because the primary ecological reactions of fish species to climate heating are migration and range shifts, current physical and chemical barriers in freshwater ecosystems will limit those processes, in some cases leading to extirpations and extinctions. There is relatively limited evidence that current extinctions result from climate heating^2^. However, climate heating may overtake habitat destruction as the chief cause of species extinction in future decades^3^. For conservation and management purposes, assessing the degree to which species will likely respond to climate heating over time is critical.

Iran, supports relatively high species richness and biodiversity of all taxonomic groups^4–7^. This Iranian biodiversity has resulted from (i) its highly diverse climate and geography, (ii) its active geological history^5, 6, 8^, and (iii) its biogeographic location at the intersection of three major biogeographic zones, i.e., Ethiopian, Palearctic, and Oriental^6, 9, 10^. Thus, although Iran is a poorly represented nation in biodiversity-climate heating studies, it is an important region because it is a biodiversity hotspot. Furthermore, endemic species losses there mean losses of species found nowhere else on Earth. Also, its biogeographic location at the intersection of three major biogeographic zones means that what we learn about fish biodiversity threats in Iran has implications for related fishes on three continents^11^.

Nonetheless, Iranian rivers are under severe human pressures from dams, agriculture, urbanization, water abstraction, channelization, overfishing, invasive non-native species, translocations of native species, and water pollution^6, 12^. However, climate heating is becoming the most important threat to Iranian fish biodiversity^12–16^. The mean temperature of Iran is predicted to rise from 1.2 to 7.8 °C by the end of the century (2080–2100) and snow cover and precipitation are predicted to decrease considerably^17, 18^. Therefore, Iran is highly vulnerable to global climate heating^19–21^.

Species distribution modeling (SDM) is an essential tool for species conservation and management because it can predict geographic distribution patterns of different species under the influence of environmental variables^22–24^. Therefore, SDM is valuable for making conservation decisions^25^. These models are particularly useful for determining factors that limit species distributions, such as the impacts of climate heating^26, 27^. SDM is being regularly used for predicting climate heating impacts on species distributions, and there is increasing evidence of its usefulness for this purpose^28, 29^. Assuming that the climatological niche of species remains the same over ecological time, we can use SDM and forecasted climate scenarios to predict future species distribution ranges^30^. SDM thus helps us estimate the degree to which future climate conditions are likely to be suitable for specific fish species. Additionally, scientists have used SDM to estimate habitat alteration effects^14, 31^, suitable habitats^32^, the probability of species invasion^33^, and the relationships between different species and their environments^34–36^.

Predicting the distribution of fish species as a result of climate heating is necessary for natural resource management in the Iranian biological hotspot. Therefore, the objectives of our study are to (i) determine the major variables influencing the different fish species’ distributions, (ii) estimate climate heating impacts on the fish species distribution in different categories (total, endemic, non-native, native, IUCN red-list), and (iii) estimate climate heating-based alterations in fish species richness patterns.

## Materials and methods

### Study area and sampling

**Figure 1:**
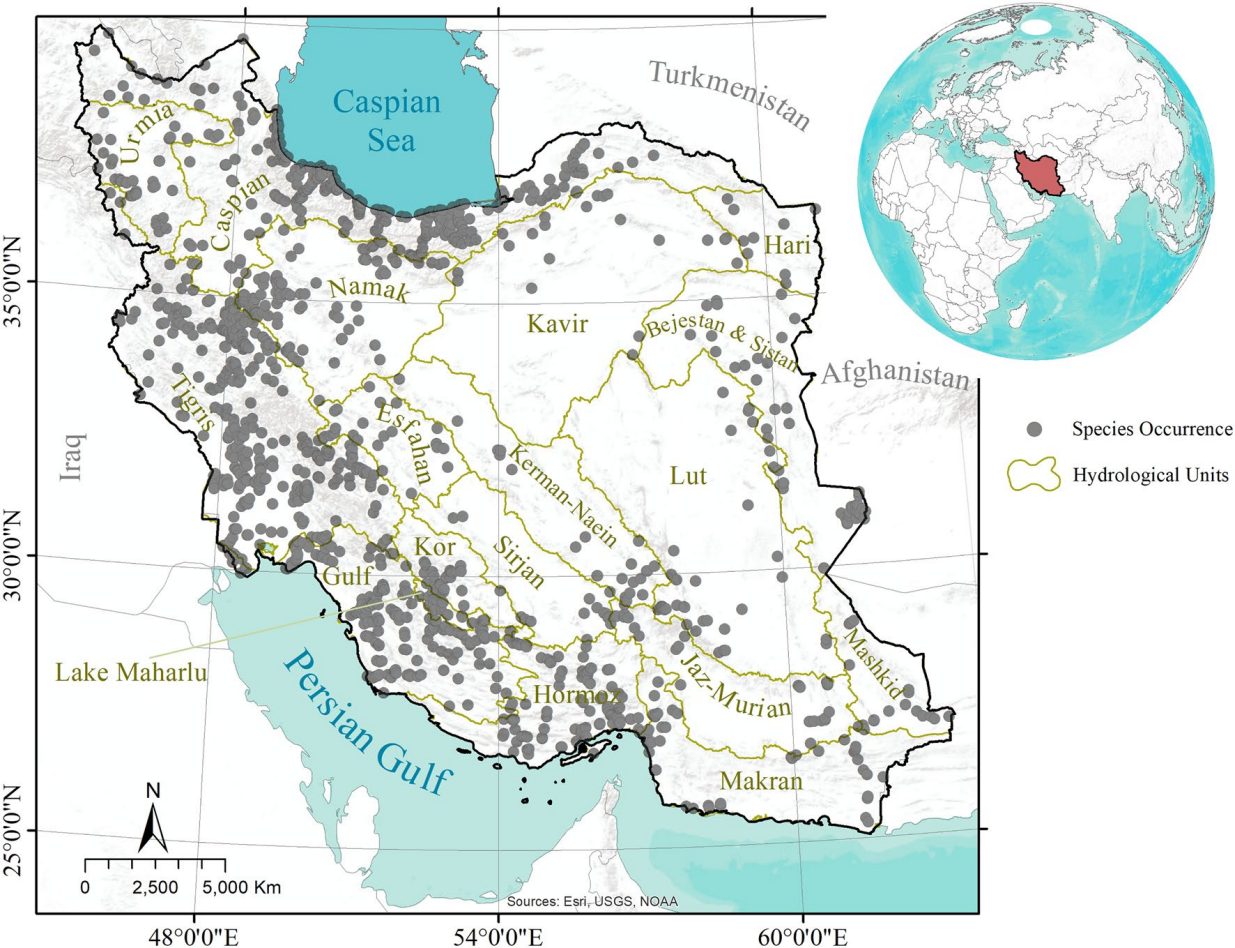
The study area and major hydrologic units in Iran (Coad, 1980).

Iran’s land area is 1,629,807 km^2^ and it ranges from latitudes of 25–40 degrees north and longitudes of 44–64 degrees east. Iran has 19 major hydrologic units^37^ (Fig. 1), all of which we address in the present study. The prevailing climate in Iran is arid and semi-arid, with a mean annual rainfall of < 250 mm in more than 80% of the nation. Also, seasonal temperatures range from − 20 to + 50 °C^6^. Our study covers all the permanent rivers of Iran —but perhaps an insufficient number of sites were sampled in all named rivers, and the sites were not selected through use of a probability sampling design. The places that are not covered are related to non-permanent rivers.

### Studied species

Among 274 potential species, 131 riverine fish species (i.e., 91 natives, 28 endemics, 12 non-natives, and 13 threatened) were selected based on the availability of data as well as their potential for modeling (see Appendix 1). The 142 rejected species were eliminated from modeling because of < 5 occurrence points. The 131 modeled species represent 32 families (with number of species per family): Cyprinidae (35), Leuciscidae (27), Gobiidae (12), Nemacheilidae (9), Aphaniidae (5), Acipenseridae (4), Cobitidae (3), Gobionidae (3), Xenocyprididae (3), Danionidae (2), Gasterosteidae (2), Mugilidae (2), Percidae (2), Salmonidae (2), Siluridae (2), Sisoridae (2), Acheilognathidae (1), Anguillidae (1), Atherinidae (1), Bagridae (1), Carcharhinidae (1), Chanidae (1), Cichlidae (1), Clupeidae (1), Esocidae (1), Heteropneustidae (1), Mastacembelidae (1), Petromyzontidae (1), Poeciliidae (1), Sparidae (1), Tincidae (1), and Syngnathidae (1).

Species occurrences were obtained from our fieldwork, previous literature, personal datasets of experts (i.e., unpublished data, not deposited into an online database), and museum data for 1481 sites with known geographical coordinates. Sites that contained a targeted fish species were marked as observed, and sampled sites without the targeted fish species were marked as background (pseudo-absence). Each species needs presence and background points for modeling. Previous studies^38, 39^ have emphasized the importance of selecting the background points for a species independently of its actual presence locations. This helps ensure that the background points provide a fair representation of the environment, rather than being influenced by the target species' distribution alone; therefore, the pseudo-locations of a species can be included in the background points^38^. The background points for each species were selected by combining its presence locations with an additional 500 selected presence points for other species^38^. This is essential for reducing sampling bias and improving SDM predictive performance.

### Environmental variables

Based on previous studies^16, 34^, nine potential natural variables known to affect fish species distribution were identified: elevation (ELE/m), maximum site river width (Max-WID/m), site average annual precipitation (PRE), site slope (SLO/%), site mean annual air temperature (A-TEM/°C), site maximum air temperature of the warmest month (Tmax/°C), site minimum air temperature of the coldest month (Tmin/°C), air temperature difference between the coldest and warmest months of the year (R-TEM/°C), and HU occupancy of the target species. The HU code (location) is a good synthetic descriptor of regional environmental constraints for fish and was retained as a dummy variable. In the next step, to avoid collinearity, we calculated Spearman correlations among the variables. We removed any variables out of a pair with a high correlation (*r* >*|*0.75*|*) as suggested by previous studies^16, 35^. We removed only Tmax and Tmin. The HUs were based on Coad^37^. All other variables were extracted and measured based on^16^.

Non-climatic (Max-WID, ELE, SLO, and HU) and climatic variables (PRE, A-TEM, and R-TEM) were obtained from Google Earth (Version 5; Google Inc.) and the WorldClim database^40^, respectively. To extract the climatic variables, we used a 5-km buffer around each sampling site^16^, and the average value of each climatic variable in those buffers was extracted. This method does not reflect the effects of catchments on sites, instead, it provides site-specific data^16^.

In the next step, the predicted climatic variables were provided for the 2050s and 2080s from the CCAFS-Climate (http://www.ccafs-climate.org) nested in two General Circulation Models (GCMs). GCMs are the major tool to provide climate heating information under different greenhouse gas emission scenarios. There are several paths to attain human radiative forcings, which represent various paths of greenhouse gas concentrations, the so-called representative concentration pathways (RCP). Among different scenarios RCP 8.5 and RCP 4.5 were used for this study. RCP 4.5 is the optimistic scenario in terms of future amounts of fossil fuel production; RCP 8.5, with the highest amount of greenhouse gas emission, is the pessimistic scenario^41^. The different RCPs can have different effects on species distributions and are associated with a degree of uncertainty in the modeling process^42^.

### Species distribution modeling

To predict fish species distributions, we used the Maximum Entropy (MaxEnt)^22^ algorithm in R programming ^43^, and the dismo package^44^. MaxEnt creates robust and accurate predictions and distribution models that are comparable to ensemble methods in terms of accuracy^45, 46^. It is a well-established modelling method with a flexible response and good transferability power^47^. These features make it a suitable model for predicting the potential distribution of species under different environmental conditions and a valuable tool for conservation and ecology applications.

The MaxEnt algorithm produces a continuous distribution map that ranges from 0 to 1, representing the relative likelihood that a species will occur or not at a given location. To create a binary prediction map of presence and absence, a threshold value must be selected that separates the continuous distribution into two classes, i.e. presence or absence^48^. To determine the threshold for generating binary habitat maps, the “equal training sensitivity and specificity” metric was used^26^. These threshold values are chosen to balance between model accuracy and overfitting^48^.

We used a tenfold cross-validation method to measure model accuracy. To do so, the dataset was randomly divided into ten parts, nine of which were used to create the model. Then the model was used to evaluate the holdout (tenth) part^20^. We used the AUC (area under the receiver operating characteristic curve (ROC)) metric to assess model accuracy^49^. The ROC curve is independent of prevalence, and AUC shows the ability of the model to discriminate presence and absence points correctly^50^. Its value ranges between 0 and 1 (indicating a perfect model) with 0.5 showing a random model. In general, AUCs > 0.7 are valid and acceptable, whereas those above 0.9 indicate excellent results^51^.

The future distribution of species was also calculated by the projection of the models for the future using both RCP 4.5 and 8.5 scenarios in the 2050s and 2080s, creating four predicted distributions for each species^42^. To assess the influence of climate heating on species distribution, we compared each future species distribution map with current distribution maps. We used binary maps created using thresholds to make the calculation of range differences easier. This allowed us to effectively identify and quantify the shifts in species distribution (e.g., each species presence point in the current map that becomes absent in the future constitutes a loss). Also, to evaluate the effects of elevation range shifts, the elevation range (0 m to 3000 m) was divided into four classess: (1) very low (up to 100m) with sea connection; (2) low without sea connection (between 100 and 500m); (3) medium (500–1500m); and (4) high altitudes (> 1500m).

The amount of transformation in each species range was computed by identifying the number of stable, lost, and gained suitable sites for all species (Table 1). To calculate fish species richness, we first summed the continuous predicted occurrence likelihood for each point. Then, for each HU, we applied a threshold to the likelihood of each point and counted the number of species occurrences within the HU. This binary data was then used to determine the species richness for each HU.

**Table 1:** Range shift calculation.

Parameter	Percent of loss	Percent of gain	Species range change
Formula	(Loss/NC*) × 100	(Gain/NC) × 100	% of Gain–% of Loss

*NC is the current distribution of each species.

The **loss** sites are those where species presence relative likelihood is less than the threshold (determined for projecting binary habitat suitability) and the **gain** sites are those where species presence relative likelihood is greater than the threshold.

## Results

### Major variables influencing species distributions

Species model discrimination assessed via AUC, ranged from 0.74 to 0.99, indicating that all models were valid, with 90 species having excellent (0.9–1), 34 species with good (0.8–0.9), and eight species with acceptable (0.7–0.8) discrimination power (Appendix 2).

The factors affecting species distribution differed among species (Table 2; Appendix 3). The majority of total species (60 out of 131, 45%) were most strongly influenced by HU. Other important factors for the 91 native species were elevation (35 species, 38%), mean annual air temperature (19 species, 21%), and average annual precipitation (12 species, 13%). Slope and the air temperature difference between the coldest and hottest months were the most important variables for only two and four species, respectively. Maximum river width was not important for any species. However, the order of variable importance was different for non-native species, with elevation being the most important factor followed by HU. Overall, HU and maximum river width were the most, and least, important variables, respectively (Table 2, Appendix 3).

**Table 2:** Overall variable importance (%) for 131 fish species.

HU	ELE	A-TEM	PRE	R-TEM	SLO	Max-WID
36.8	24.1	14.0	13.2	5.5	4.1	2.1

HU: Hydrological Unit, ELE: Elevation, A-TEM: Mean annual air temperature, R-TEM: Air temperature difference between the coldest and warmest months of the year, PRE: Precipitation, SLO: Slope and Max-WID: Maximum width.

### Impacts of climate heating on fish species distribution

**Figure 2:**
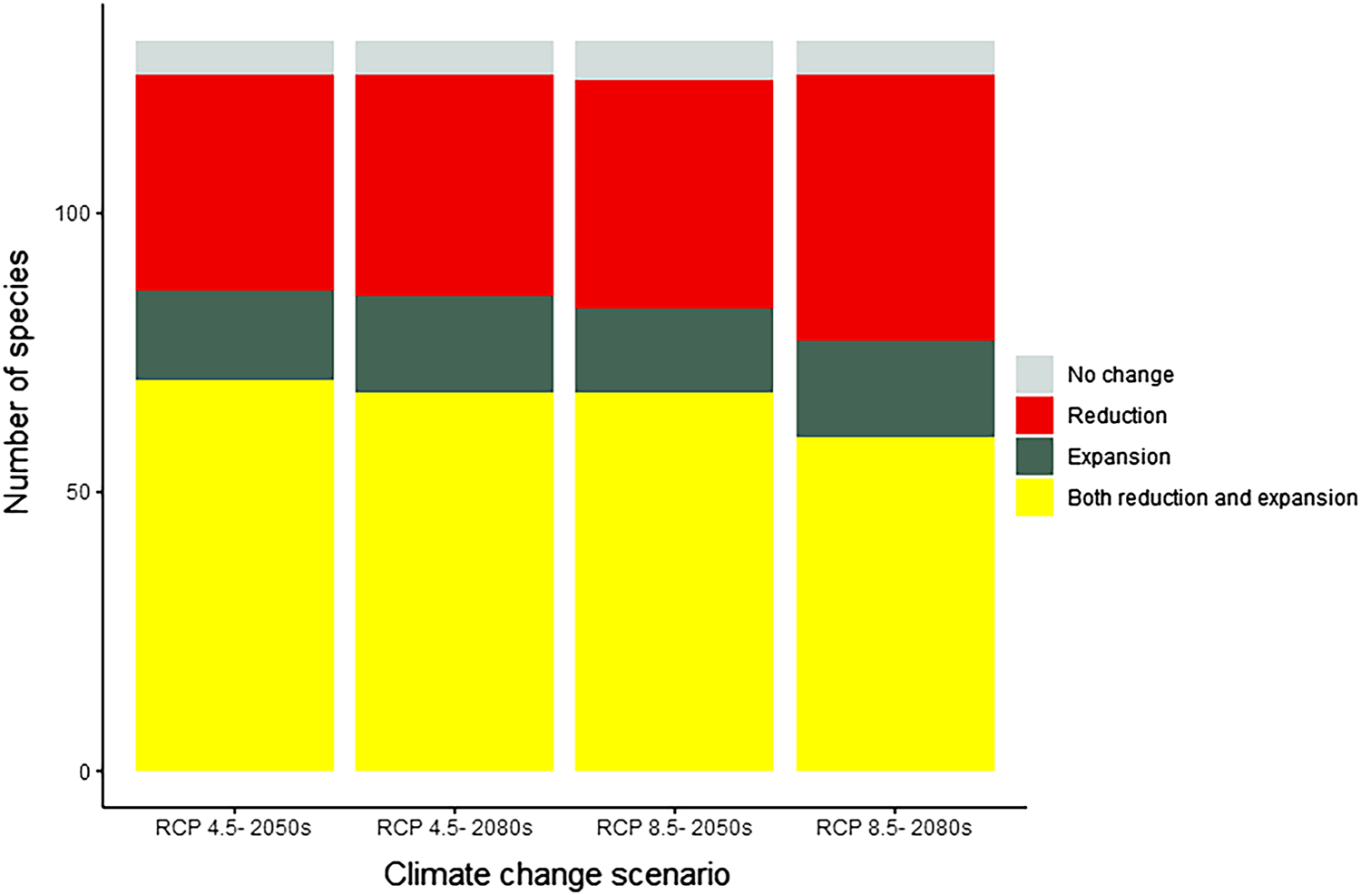
Number of species with habitats that are predicted to either change or remain nearly stable in the future.

Our results have shown that many species will react differently in the advent of future climate heating (Fig. 2; Table 3; Apppendix 2). The probable impacts of the four different climate heating scenarios on the habitat distribution of the 131 different fish species were predicted to vary across different species and scenarios (Fig. 2). For example, the distributions of 58 of the 131 species are predicted to both increase and decrease in all four climate change scenarios, indicating that these species may be particularly vulnerable to changes in their environment. Figure 2 also indicates that 37 species are predicted to experience declines in their habitat distributions, whereas 14 species are predicted to experience expanded habitat distributions. The remaining six species are predicted to show no change in their habitat distributions.The figure also could be useful for identifying species that are particularly vulnerable to climate heating.

**Table 3:** Number of fish species reacting differently to climate scenarios.

Climate scenario	Expansion	Reduction	Both expansion and reduction	No change
RCP-4.5 in 2050s	16	39	71	6
RCP-4.5 in 2080s	17	40	69	6
RCP-8.5 in 2050s	15	41	69	7
RCP-8.5 in 2080s	17	48	61	6

Regarding fish families, there are five (Syngnathidae, Anguillidae, Gasterosteidae, Salmonidae, and Tincidae) whose habitats will only decline in the future (Appendix 2). Also, Clupeidae and Cobitidae resulted in a reduction in three of the four climatic scenarios. On the other hand, our models predicted that three families (Cichlidae, Carcharhinidae, and Petromyzontidae) will expand their ranges. Moreover, the distribution of 20 fish families are predicted to both increase and decrease in comparison to their original ranges in all four scenarios(Appendix 2).

**Figure 3:**
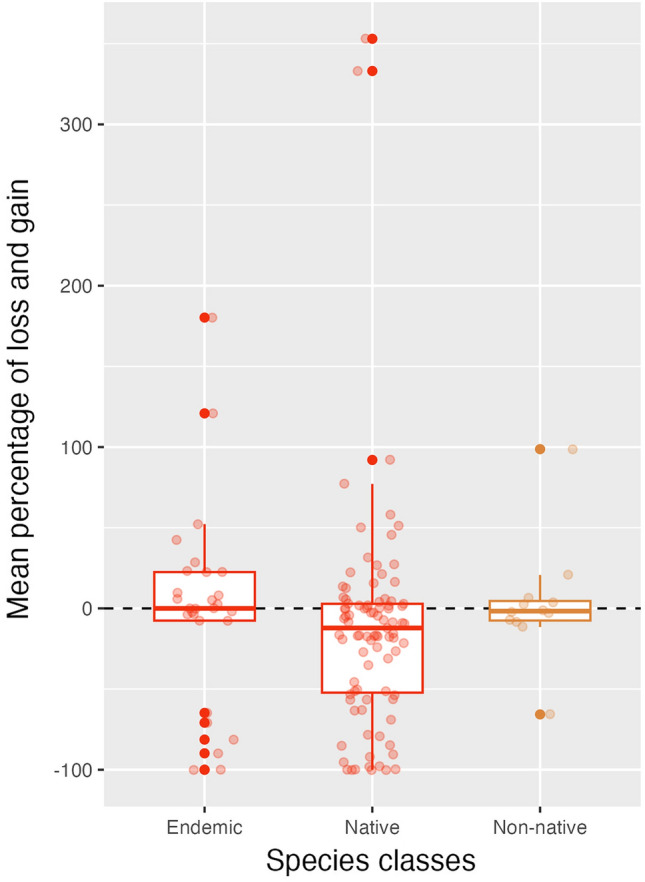
Percentages of losses and gains in the ranges of endemic, non-native, and native fish species. Box plot components are the median (middle line), the first and third quartiles (box top/bottom lines), and the vertical lines extending from the box plot lines are 1.5 * inter-quartile ranges of “mean percentage of loss and gain”. The dots refer the data that were used for creating box plots, scattered randomly (over the x-axis) and they are transparent. Darker points indicate that several points for that value overlapped.

Furthermore, all endemic and native species belonging to the families Atherinidae, Bagridae, Esocidae, Syngnathidae, Clupeidae, Cobitidae, Salmonidae, and Tincidae will be highly vulnerable to climate heating as our models showed range declines in all four scenarios (Fig. 3). Out of 28 endemic species, the distribution of four (14%), seven (25%), and 12 (43%) will decrease, increase, or show both responses in all four scenarios in comparison to their original ranges, respectively. *Alburnus doriae* and *Alburnoides petrubanarescui* will not shift at all and three other species will respond differently to each RCP scenario. In terms of non-native species, the distribution of eight of the twelve species will experience both expansion and contraction compared with their original ranges. Although three species (25%) will reduce their ranges, none will increase their ranges and a single non-native species will not change its range under any of the scenarios (Fig. 3).

**Figure 4:**
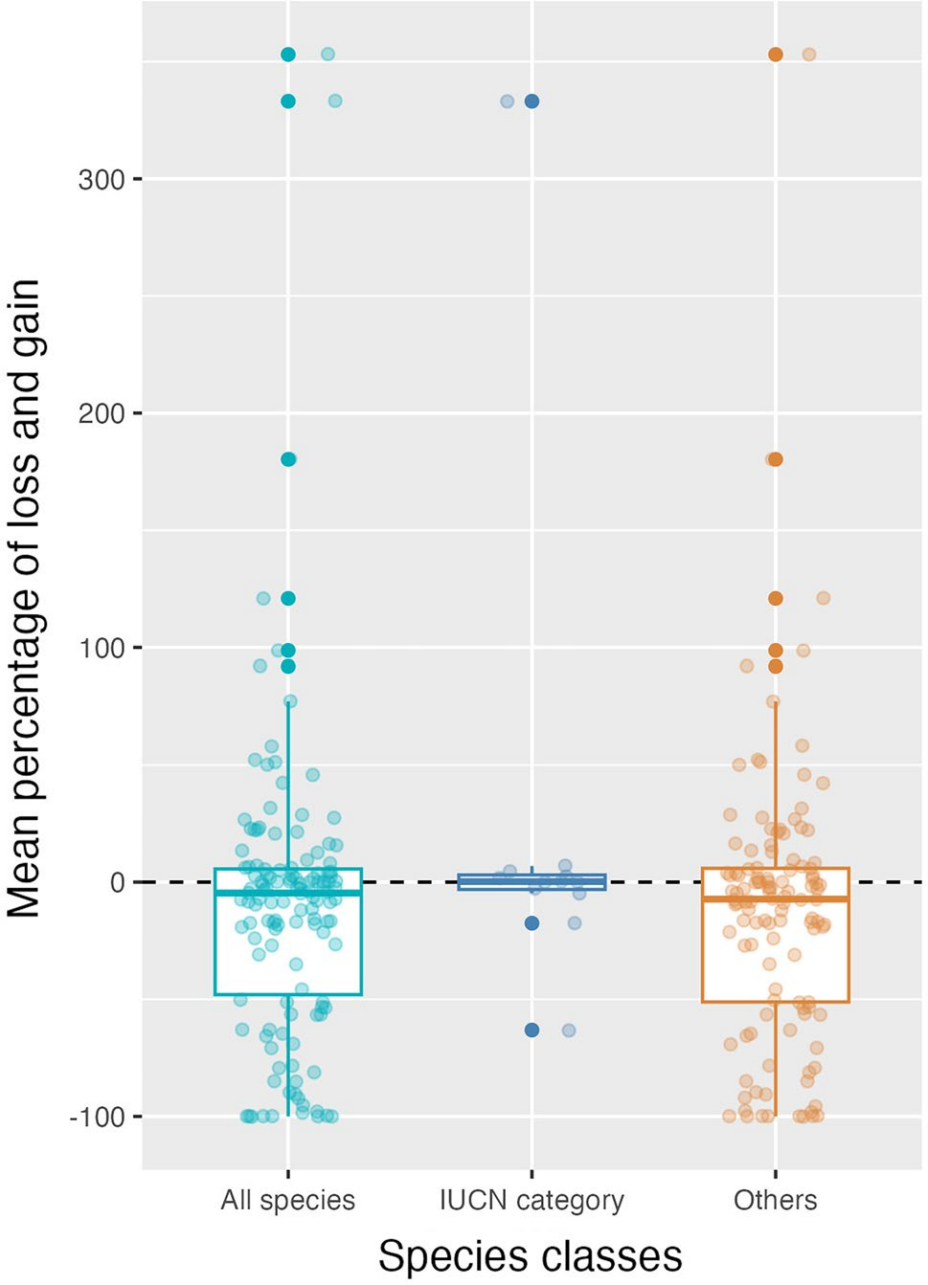
Percentages of losses and gains in the distributions for all species, IUCN red list species, and other species. Box plot components are the median (middle line), the first and third quartiles (box top/bottom lines), and the vertical lines extending from the box plot lines are 1.5 * inter-quartile ranges of “mean percentage of loss and gain”. The dots refer to the data that were used for creating box plots, scattered randomly (over the x-axis) and they are transparent. Darker points indicate that several points for that value overlapped.

Thirteen threatened species were included in this study. Five (38%) are predicted to expand their distributions, whereas 3 (23%) will face habitat loss in all scenarios and 3 (23%) others will both increase and decrease (Fig. 4).

The negative impacts of climate heating are predicted to be more severe for native than for non-native fish species (Fig. 3). There are few species whose ranges will increase more than 100%, whereas 13 are predicted to lose over 90% of their ranges in the future (*Schizocypris altidorsalis*, *Capoeta capoeta*, *Mesopotamichthys sharpeyi*, *Schizothorax zarudnyi*, *Romanogobio macropterus*, *Sabanejewia caspia*, *Paracobitis vignai*, *Salmo caspius*, *Neogobius melanostomus*, *Ponticola cyrius*, *Ponticola gorlap*, *Aphanius sophiae*, *Perca fluviatilis*). Among these species, seven are predicted to completely lose their ranges (*Schizocypris altidorsalis, Schizothorax zarudnyi, Paracobitis vignai, Perca fluviatilis, Aphanius sophiae, Aphanius farsicus, Salmo caspius*) and are at high risk of extirpation in Iran as a result of climate change.

Based on RCP 8.5 in the 2080s, the number of species losing part or all of their habitats is at its maximum (i.e., 48), whereas the lowest number of species (i.e., 39) occurs in the 2050s at RCP 4.5. The number of species expanding their ranges was very close in all scenarios (i.e., 15–17). Finally, six or seven species were predicted to be tolerant/resistant to any change under the four future scenarios (Table 3).

### Climate heating-based alterations in fish species richness

**Figure 5:**
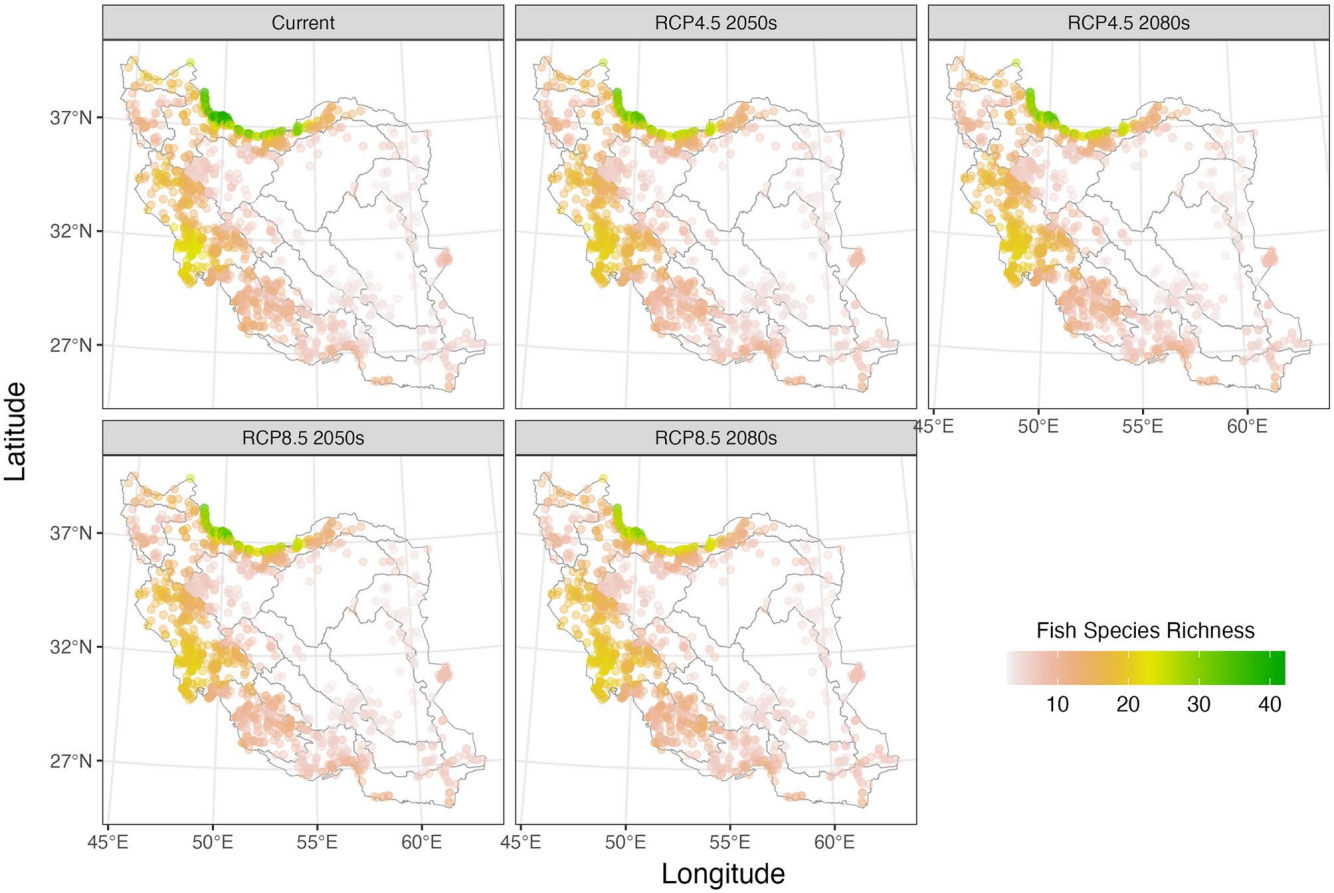
Fish species richness in sites at current and different future scenarios per HU.

Fish species richness in various sites within each hydrological unit, both in current and future scenarios differs as expected (Fig. 5; Table 4; Apppendix 5). The hydrological units with the highest fish species richness are generally in the Caspian and Tigris HUs, respectively, in both current and future scenarios. However, when considering the impacts of climate heating under optimistic and pessimistic scenarios for the 2050s and 2080s, the maximum species richness in some hydrological units is predicted to decrease (e.g. Bejestan and Sistan, Caspian, Tigris), increase (e.g. Esfahan, Kerman-Naein, Makran), or remain constant (e.g. Hari, Kavir, Namak) compared to the current scenario (Table 4; Apppendix 5). But these variations in species richness vary across different scenarios within some hydrological units (e.g., Gulf, Jaz-Murian, Urmia, Mashkid, Caspian, Tigris) (Table 4; Apppendix 5).

**Table 4:**
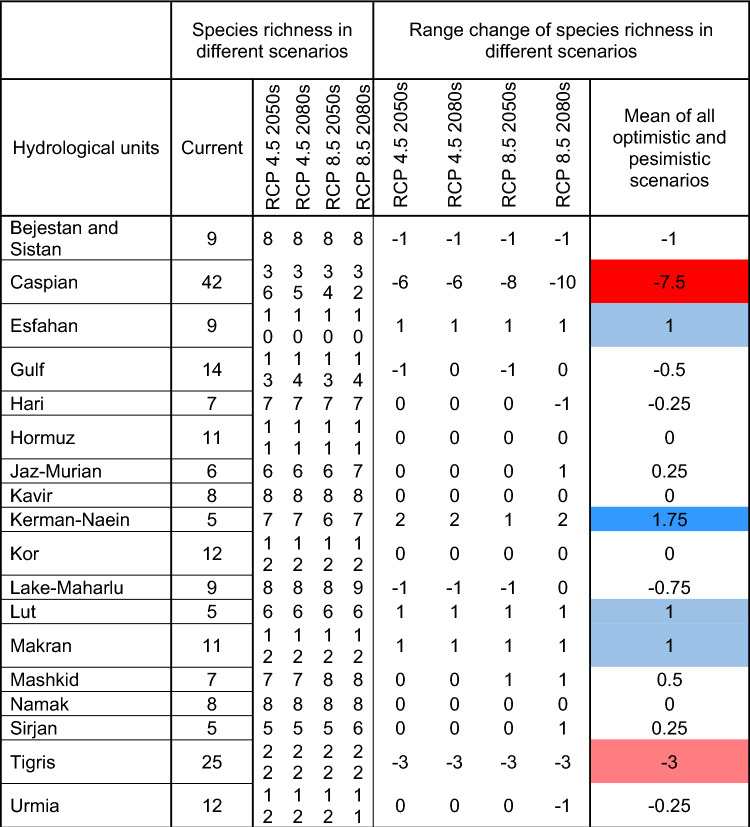
Fish species richness in different hydrologic units at current and future climate scenarios predicted for two time periods (2050s and 2080s) based on RCP 4.5 and RCP 8.5 scenarios. Red shows reduced richness; blue shows increased richness.

**Figure 6:**
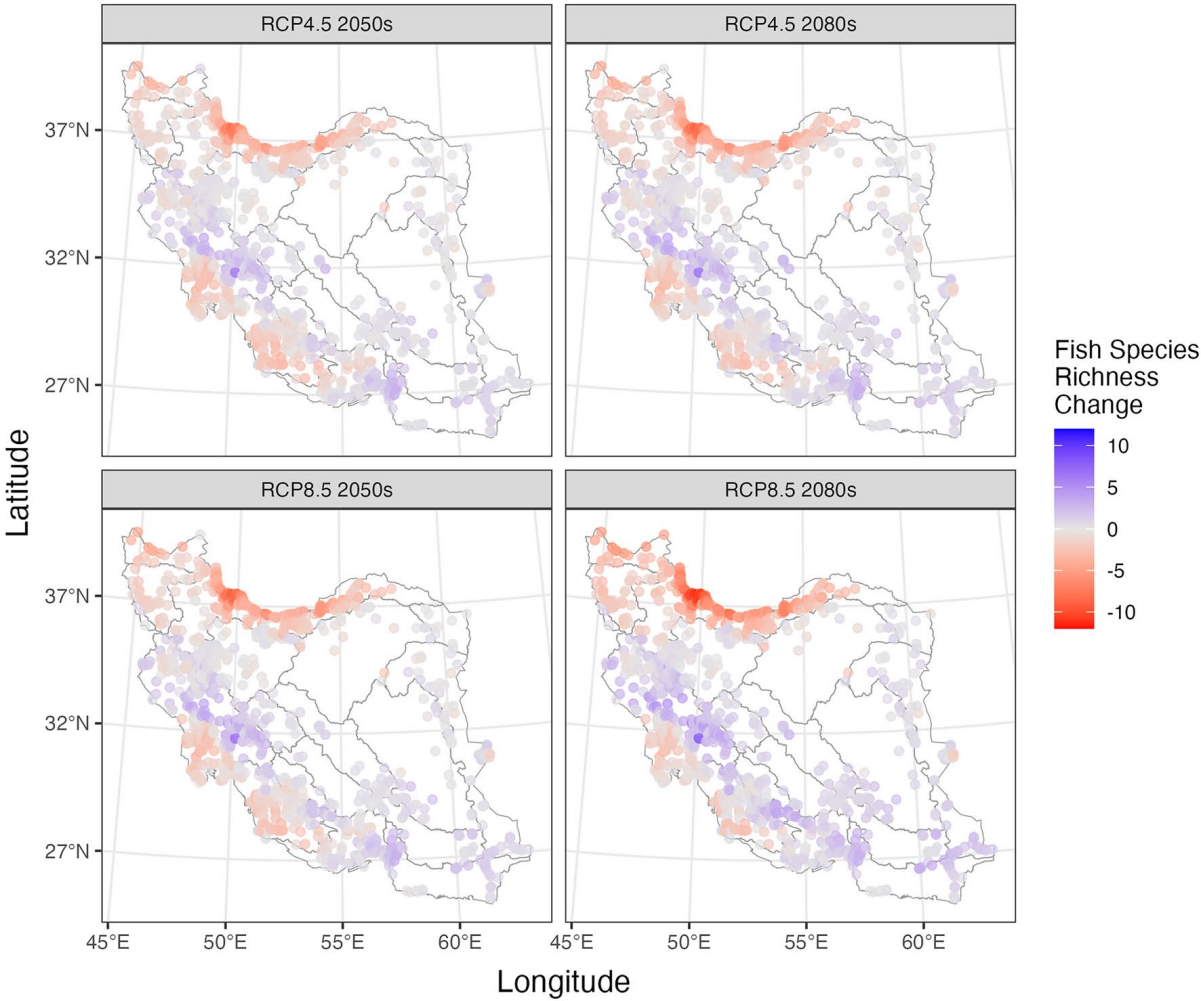
Fish species richness change in sites at different future scenarios per HU.

The predicted range changes of fish species richness differ across various sites within each hydrological unit, considering future climate scenarios (Fig. 6; Apppendix 6). Some expand ranges, others are predicted to experience range contractions, and some are likely to remain constant (Fig. 6; Apppendix 6). The Caspian and Tigris hydrological units are projected to experience the greatest decreases in fish species richness, whereas the Lut, Makran, and Esfahan units are expected to have the least increases (Table 4; Apppendix 6). Several hydrological units are predicted to experience minimal positive or negative changes in fish species richness (Table 4: "Mean of all optimistic and pessimistic scenarios" column).

**Figure 7:**
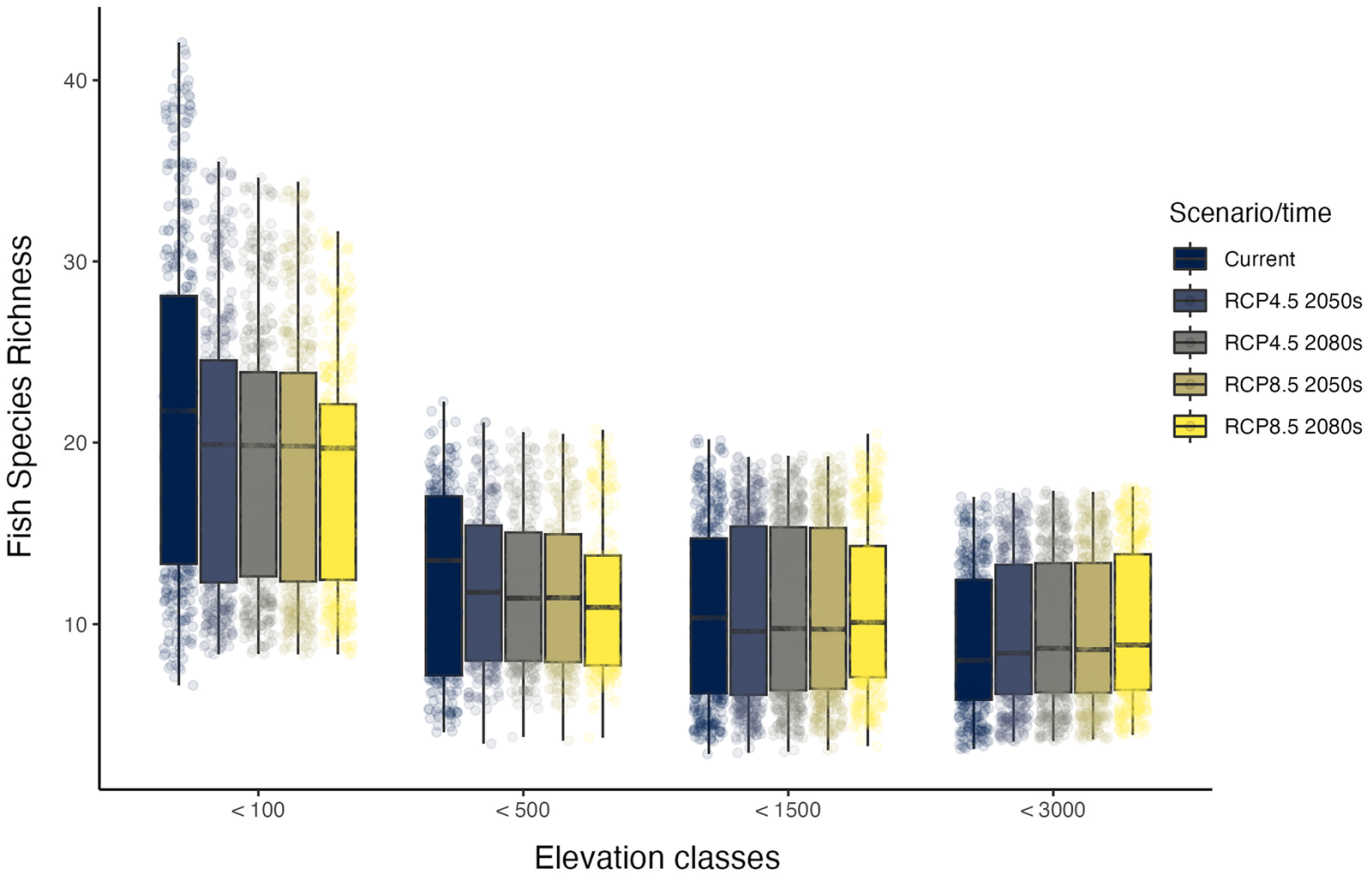
Elevation categories and fish species richness in different scenarios.

Species richness in future scenarios is predicted to decrease to some extent in comparison to current scenario in two elevation levels (i.e. < 100m and from 100 to 500m; Fig. 7). But at higher elevations (> 500), species richness may increase in some future scenarios in comparison with current conditions. This likely would result from species shifts to higher elevation because of gains in the numbers of warmer sites.

## Discussion

Modeling helps us to become more aware of areas that a species will likely occupy or any currently occupied areas from which a species will likely be lost in the future. These estimates are potentially helpful for management and conservation^35^. In general, our results are best seen as the magnitude of climate heating impacts on the distribution of 131 riverine Iranian fish species and their implications for conservation. We predicted that climate heating will cause a shift in habitat suitability by the 2050s and 2080s for most of the studied fish species and that the greatest reduction in species richness will be in the Caspian and Tigris HUs in Iran. Therefore, this study offers conservationists and management authorities valuable information about the likely future distributions of many fish species in Iranian rivers, the likely impacts of climate heating on their habitats, and the sensitivity of endemic, threatened, and non-native species to climate heating.

Climate heating has different influences on different organisms^52, 53^. In other words, some species track the same climate conditions elsewhere and others stay and either adapt or face extinction. Also, those species that shift their ranges will not react similarly either. The size of their new ranges can be the same, increase, or decrease^54, 55^. Our results revealed that the number of species confronting habitat loss in the future is higher than the number of species that will gain suitable habitats (Table 3). Moreover, according to our findings, the mean change of the habitat shift for most of the species in the future will be negative (Fig. 4). Similar conclusions have been reached in other studies. For example, in Europe, fish species are at high risk of losing their potential habitats by the 2050s (median loss: 43%), and 8 species will completely lose their ranges over time as a result of climate heating^56^. Also, a global study of freshwater fish species showed that half of the current freshwater fish species may become extinct in the next few decades^57^. Finally, we found that some endemic species are likely to disappear from Iranian waters, while other tolerant/resistant species are likely to expand their ranges, i.e., our fish fauna will become increasingly homogeneous and less regionally diverse, as predicted globally^58^.

Reduced species habitat extent is associated with increased extinction risk^59^. This is an additional threat to threatened and endemic species^60^. Therefore, it is imperative to plan conservation strategies to mitigate the impacts of climate heating and other human-caused pressures on these fish species^6, 16^.

According to our results, out of 19 HUs, 17 are going to have new fish species in the future (Appendix 4), and/or new areas in the same HU will be provided. However, many of these HUs are not connected hydrologically, so fish movement is not possible because of physical barriers.

There are at least two concerns when new HUs become suitable for a species. The species may be unable to move to new locations because of physical barriers and a lack of connectivity. In this case, a possible action is the translocation of the species to the new climatically suitable area^61^. In doing so, conservationists must pay attention to critical considerations such as local habitat suitability, disease introduction, and competition among the species^19^. The second concern is that some species can migrate to new HUs or new areas in the same HU, but the area might be impaired by anthropogenic factors (dams, non-native species, land use, water extraction, water pollution, and physical habitat degradation). Therefore, it is necessary to investigate the suitability of the new area for the species. In response to changing conditions, species will attempt to adapt or migrate, but if barriers and habitat degradation are not addressed, they are likely to become extinct or extirpated^62^.

Different species require different habitats. Iran is an environmentally heterogeneous country. Consequently, it supports relatively high species richness for an arid/semi-arid nation^4, 6, 15, 16^. We used two types of predictor variables, local (e.g., channel slope, maximum river width, elevation) and regional (HUs, climatic variables) in this study. Both categories were important in determining a fish species’ potential habitat.

Elevation was the most important local variable by far in our study. It was also crucial in determining fish species habitat suitability in other studies^12–14, 63^. However, we observed no correlation between elevation and temperature across the extent of Iran, perhaps because of elevation co-varying with precipitation, and river size.

Temperature and precipitation were important regional variables in our study. In many other studies, temperature was a key factor in fish species occurrences^16, 65^. Because freshwater fishes are ectotherms, they are particularly sensitive to temperature for their metabolism, development, growth, and reproduction^64^. Precipitation along with elevation and slope affect current velocities and create various habitats for fish species^66^. These variables were also important in several other studies for determining many fish species’ habitat ranges^11–13, 16, 34^.

Based on our results, most areas with a high likelihood of fish species presence were in regions with high precipitation and in some co-occurring anthropogenically impaired areas. In other words, fish and humans both need water. However, species distributions will not remain static because environmental conditions are constantly changing, even more so with climate heating^67^. For conservation purposes, it is important to anticipate future shifts in fish distribution as many of their potential habitats change. The greatest reduction in species richness is predicted for the Caspian and Tigris HUs which are the regions with the greatest precipitation in the country. Others have also predicted that the greatest declines in fish species richness are likely to occur in basins with greater precipitation and warmer temperatures^56^.

Two studies diagnosed multiple threats to Iranian rivers and fishes (agriculture, urbanization, water and sand extraction, channelization, fragmentation by dams, water pollution, unregulated fishing, and non-native species)^6, 16^. Climate heating is one more major pressure on fish habitats and increases extinction risks. However, by rehabilitating degraded habitats, increasing river connectivity, protecting key areas, and relocating populations into new potential habitats, we may be able to mitigate the impacts of climate heating on fish species.

It is also important to indicate that using SDMs helps us to broaden our knowledge about the threats to fish species and their potential future ranges, species richness, and species composition. SDM can be extremely useful for designing conservation and management strategies and priorities^68, 69^. However, the sources of uncertainty must be considered carefully. Climate heating projections deal with some issues such as greenhouse gas emission scenarios and modeling uncertainty as well as natural variability^70, 71^. Another critically important set of variables are the human activities that were not addressed in our study. These factors can accelerate or slow the negative effects of climate heating^72^. Human activities can either accelerate or slow the effects of climate heating. For example, burning fossil fuels leads to global heating^73^. Afforestation and reforestation efforts remove carbon dioxide from the atmosphere and slow the effects of climate heating as can reduce greenhouse gas emissions to sequester carbon dioxide from the atmosphere and mitigate the effects of climate heating^74–76^. Furthermore, data on sub-catchments and ecoregions, which were not available in Iran during our study^12–16^ can further reduce uncertainty. Therefore, we believe that other studies considering those omitted elements may show slightly different results.

## Conclusions

Results revealed that the southern Caspian and Tigris HUs in Iran will be at higher risk for reduced species richness from climate heating. To mitigate climate heating impacts in those regions, it is crucial to reduce the human pressures from land use, river fragmentation, and impaired hydromorphology and water quality. A number of strategies should be considered: stakeholder education, designation of protected rivers, barrier removal, and riparian shading. The current fish species richness in Iranian rivers can be used to help select protected areas for freshwater ecosystems. This is an essential step in preserving Iranian ichthyodiversity. There are over 650 major dams in Iran, thus limiting migration barriers is key to facilitating fish movements. To address this issue, the implementation of efficient fishways is crucial. Land uses and water abstractions are major causes of water pollution and altered flows. Riparian protections are a cost-effective strategy for mitigating catchment land uses by providing shade, wood debris, and sediment reduction^73^. Markedly improved water conservation is critical for an arid/semi-arid nation like Iran; therefore, defining and implementing environmenthal flows are necessary steps. Together, those actions will help to create a more resilient and adaptable ecosystem that is better able to withstand the effects of climate heating and provide critical ecosystem services for humans.

It is important to consider regional spatial extents as well as local conditions when assessing community dynamics and trophic interactions^1^. Future studies should include more comprehensive datasets. Although climate heating is a clear threat to fish species persistence, other anthropogenic threats must be considered as well to ensure successful fish species conservation. Finally, to prevent Iranian fish species from extinction in the face of rapid climate heating, it is necessary for these fish species be able to keep pace with the new changing conditions. The most effective way of achieving this goal is to slow the rate of climate heating by reducing global greenhouse gas emissions^77^.

### Supplementary Information

Supplementary Information 1.

## Data Availability

The datasets used and/or analyzed during the current study are available from the corresponding author upon reasonable request.
