# Supplementary material for: Predicting climate heating impacts on riverine fish species diversity in a biodiversity hotspot region
Source: Sci Rep. 2023 Sep 1;13:14347. doi: 10.1038/s41598-023-41406-9 (PMC10474041; doi:10.1038/s41598-023-41406-9)

# Appendix 1. The number of endemic, non-native, and IUCN-red-list species (CR, EN, VU, NT, LC, DD).

| **Row** | **Family** | **Endemic** | **Non-native** | **CR** | **EN** | **VU** | **NT** | **LC** | **DD** |
| --- | --- | --- | --- | --- | --- | --- | --- | --- | --- |
| 1 | Petromyzontidae | - | - | - | - | - | 1 | - | - |
| 2 | Carcharhinidae | - | - | - | - | - | 1 | - | - |
| 3 | Acipenseridae | - | - | 4 | - | - | - | - | - |
| 4 | Anguillidae | - | 1 | 1 | - | - | - | - | - |
| 5 | Clupeidae | - | - | - | - | - | - | 1 | - |
| 6 | Chanidae | - | - | - | - | - | - | 1 | - |
| 7 | Leuciscidae | 8 | - | - | - | - | - | 16 | - |
| 8 | Cyprinidae | 8 | 3 | 1 | - | 7 | - | 12 | - |
| 9 | Danionidae | - | - | - | - | - | - | 2 | - |
| 10 | Xenocyprididae | - | 3 | - | - | - | 1 | 2 | - |
| 11 | Gobionidae | 1 | 1 | - | - | - | - | 1 | - |
| 12 | Acheilognathidae | - | - | - | - | - | - | 1 | - |
| 13 | Tincidae | - | - | - | - | - | - | 1 | - |
| 14 | Cobitidae | 1 | - | - | - | - | - | 1 | - |
| 15 | Nemacheilidae | 4 | - | - | - | - | - | 2 | - |
| 16 | Bagridae | - | - | - | - | - | - | 1 | - |
| 17 | Siluridae | - | - | - | - | - | - | 2 | - |
| 18 | Sisoridae | 1 | - | - | - | - | - | - | 1 |
| 19 | Heteropneustidae | - | 1 | - | - | - | - | 1 | - |
| 20 | Salmonidae | - | - | - | - | - | - | 1 | - |
| 21 | Esocidae | - | - | - | - | - | - | 1 | - |
| 22 | Gobiidae | - | - | - | - | - | - | 8 | - |
| 23 | Mugilidae | - | 1 | - | - | - | - | 2 | - |
| 24 | Cichlidae | 1 | - |  |  |  |  |  |  |
| 25 | Atherinidae | - | - | - | - | - | - | 2 | - |
| 26 | Cyprinodontidae | 4 | - | - | - | - | - | 2 | - |
| 27 | Poeciliidae | - | 1 | - | - | - | - | 1 | - |
| 28 | Mastacembelidae | - | - | - | - | - | - | 1 | - |
| 29 | Syngnathidae | - | - |  |  |  |  |  | 1 |
| 30 | Percidae | - | - | - | - | - | - | 2 | - |
| 31 | Gasterosteidae | - | 1 | - | - | - | - | 2 | - |
| 32 | Sparidae | - | - | - | - | - | - | 1 | - |

# Appendix 2. Future percentage of Reduction/ Expansion/ Range changes of fish species in each of the time periods (2050 and 2080) based on RCP 4.5 and RCP 8.5 scenarios. △: IUCN category, 🞎: non-native, *: endemic

| **Species** | **RCP4.5** | | | **RCP8.5** | | **AUC** |
| --- | --- | --- | --- | --- | --- | --- |
|  |  | **2050** | **2080** | **2050** | **2080** |  |
| **Petromyzontidae** |  |  |  |  |  |  |
| *Caspiomyzon wagneri*^△^ | Expansion(%( | 1.84 | 1.84 | 1.84 | 1.84 | 0.93 |
|  | Reduction (%) | 0 | 0 | 0 | 0 |  |
|  | Species range changes(%) | 1.84 | 1.84 | 1.84 | 1.84 |  |
| **Carcharhinidae** |  |  |  |  |  |  |
| *Carcharhinus leucas*^△^ | Expansion | 308.11 | 321.62 | 329.73 | 372.97 | 0.978 |
|  | Reduction | 0 | 0 | 0 | 0 |  |
|  | Range change | 308.11 | 321.62 | 329.73 | 372.97 |  |
| **Acipenseridae** |  |  |  |  |  |  |
| *Acipenser gueldenstaedtii*^△^ | Expansion | 0 | 0 | 0 | 0 | 0.965 |
|  | Reduction | 14.94 | 17.24 | 17.24 | 20.69 |  |
|  | Range change | -14.94 | -17.24 | -17.24 | -20.69 |  |
|  |  |  |  |  |  |  |
| *Acipenser persicus*^△^ | Expansion | 9.82 | 9.20 | 9.20 | 10.43 | 0.959 |
|  | Reduction | 4.29 | 3.68 | 3.07 | 0 |  |
|  | Range change | 5.52 | 5.52 | 6.13 | 10.43 |  |
|  |  |  |  |  |  |  |
| *Acipenser stellatus*^△^ | Expansion | 5.99 | 6.59 | 5.99 | 4.79 | 0.957 |
|  | Reduction | 4.79 | 4.79 | 4.79 | 7.19 |  |
|  | Range change | 1. 20 | 1.80 | 1.20 | -2.40 |  |
|  |  |  |  |  |  |  |
| *Huso huso*^△^ | Expansion | 0 | 0 | 0 | 0 | 0.974 |
|  | Reduction | 2.63 | 2.63 | 2.63 | 2.63 |  |
|  | Range change | -2.63 | -2.63 | -2.63 | -2.63 |  |
| **Anguillidae** |  |  |  |  |  |  |
| *Anguilla anguilla*^△🞎^ | Expansion | 0 | 0 | 0 | 0 | 0.973 |
|  | Reduction | 2.37 | 2.84 | 2.84 | 3.79 |  |
|  | Range change | -2.37 | -2.84 | -2.84 | -3.79 |  |
| **Clupeidae** |  |  |  |  |  |  |
| *Tenualosa ilisha* | Expansion | 0 | 0 | 0 | 41.79 | 0.929 |
|  | Reduction | 60.45 | 63.43 | 61.94 | 57.46 |  |
|  | Range change | -60.45 | -63.43 | -61.94 | -15.67 |  |
| **Chanidae** |  |  |  |  |  |  |
| *Chanos chanos* | Expansion | 82.72 | 90.12 | 80.25 | 62.96 | 0.917 |
|  | Reduction | 1.23 | 1.23 | 1.23 | 3.70 |  |
|  | Range change | 81.48 | 88.89 | 79.01 | 59.26 |  |
|  |  |  |  |  |  |  |
| **Leuciscidae** |  |  |  |  |  |  |
| *Abramis brama* | Expansion | 0.67 | 0.67 | 0.67 | 0 | 0.956 |
|  | Reduction | 26.67 | 32.00 | 32.67 | 50.67 |  |
|  | Range change | -26.00 | -31.33 | -32.00 | -50.67 |  |
|  |  |  |  |  |  |  |
| *Acanthobrama marmid* | Expansion | 33.64 | 35.51 | 35.51 | 47.20 | 0.911 |
|  | Reduction | 11.68 | 6.54 | 6.54 | 0.93 |  |
|  | Range change | 21.96 | 28.97 | 28.97 | 46.26 |  |
|  |  |  |  |  |  |  |
| *Acanthobrama microlepis* | Expansion | 64.55 | 68.25 | 69.31 | 83.60 | 0.939 |
|  | Reduction | 20.11 | 16.40 | 15.87 | 1.06 |  |
|  | Range change | 44.44 | 51.85 | 53.44 | 82.54 |  |
|  |  |  |  |  |  |  |
| *Acanthobrama persidis** | Expansion | 49.25 | 77.61 | 68.66 | 114.93 | 0.954 |
|  | Reduction | 25.37 | 25.37 | 25.37 | 25.37 |  |
|  | Range change | 23.88 | 52.24 | 43.28 | 89.55 |  |
|  |  |  |  |  |  |  |
| *Acanthobrama urmianus** | Expansion | 4.00 | 8.00 | 8.00 | 12.00 | 0.988 |
|  | Reduction | 0 | 0 | 0 | 0 |  |
|  | Range change | 4.00 | 8.00 | 8.00 | 12.00 |  |
|  |  |  |  |  |  |  |
| *Alburnoides eichwaldii* | Expansion | 0 | 0 | 0 | 0 | 0.835 |
|  | Reduction | 73.61 | 83.33 | 83.33 | 100 |  |
|  | Range change | -73.61 | -83.33 | -83.33 | -100 |  |
|  |  |  |  |  |  |  |
| *Alburnoides namaki** | Expansion | 12.24 | 13.27 | 13.27 | 14.29 | 0.953 |
|  | Reduction | 7.14 | 7.14 | 10.20 | 37.76 |  |
|  | Range change | 5.10 | 6.12 | 3.06 | -23.47 |  |
|  |  |  |  |  |  |  |
| *Alburnoides petrubanarescui** | Expansion | 0 | 0 | 0 | 0 | 0.99 |
|  | Reduction | 0 | 0 | 0 | 0 |  |
|  | Range change | 0 | 0 | 0 | 0 |  |
|  |  |  |  |  |  |  |
| *Alburnoides qanati** | Expansion | 0 | 0 | 0 | 0 | 0.987 |
|  | Reduction | 73.33 | 66.67 | 73.33 | 70 |  |
|  | Range change | -73.33 | -66.67 | -73.33 | -70 |  |
|  |  |  |  |  |  |  |
|  |  |  |  |  |  |  |
| *Alburnus chalcoides* | Expansion | 13.73 | 16.18 | 16.67 | 18.14 | 0.896 |
|  | Reduction | 2.45 | 2.45 | 2.45 | 2.94 |  |
|  | Range change | 11.27 | 13.73 | 14.22 | 15.20 |  |
|  |  |  |  |  |  |  |
| *Alburnus doriae** | Expansion | 0 | 0 | 0 | 0 | 0.99 |
|  | Reduction | 0 | 0 | 0 | 0 |  |
|  | Range change | 0 | 0 | 0 | 0 |  |
|  |  |  |  |  |  |  |
| *Alburnus filippii* | Expansion | 9.78 | 8.70 | 8.70 | 2.17 | 0.943 |
|  | Reduction | 55.98 | 63.04 | 62.50 | 73.91 |  |
|  | Range change | -46.20 | -54.35 | -53.80 | -71.74 |  |
|  |  |  |  |  |  |  |
| *Alburnus hohenackeri* | Expansion | 2.68 | 3.02 | 3.02 | 3.69 | 0.885 |
|  | Reduction | 0.34 | 0.34 | 0.34 | 0.34 |  |
|  | Range change | 2.35 | 2.68 | 2.68 | 3.36 |  |
|  |  |  |  |  |  |  |
| *Alburnus sellal* | Expansion | 1.69 | 1.69 | 1.69 | 1.69 | 0.84 |
|  | Reduction | 16.18 | 17.63 | 18.36 | 22.46 |  |
|  | Range change | -14.49 | -15.94 | -16.67 | -20.77 |  |
|  |  |  |  |  |  |  |
| *Alburnus ulanus** | Expansion | 30.43 | 30.43 | 30.43 | 13.04 | 0.981 |
|  | Reduction | 13.04 | 26.09 | 26.09 | 39.13 |  |
|  | Range change | 17.39 | 4.35 | 4.35 | -26.09 |  |
|  |  |  |  |  |  |  |
| *Blicca bjoerkna* | Expansion | 0 | 0 | 0 | 0 | 0.943 |
|  | Reduction | 57.85 | 74.38 | 80.99 | 100 |  |
|  | Range change | -57.85 | -74.38 | -80.99 | -100 |  |
|  |  |  |  |  |  |  |
| *Chondrostoma orientale** | Expansion | 0 | 0 | 0 | 0 | 0.991 |
|  | Reduction | 72.73 | 45.45 | 72.73 | 68.18 |  |
|  | Range change | -72.73 | -45.45 | -72.73 | -68.18 |  |
|  |  |  |  |  |  |  |
| *Chondrostoma regium* | Expansion | 0.59 | 0.30 | 0.30 | 0.89 | 0.843 |
|  | Reduction | 2.97 | 5.04 | 3.86 | 7.42 |  |
|  | Range change | -2.37 | -4.75 | -3.56 | -6.53 |  |
|  |  |  |  |  |  |  |
| *Leuciscus aspius* | Expansion | 0 | 0 | 0 | 0 | 0.958 |
|  | Reduction | 56.79 | 61.73 | 62.96 | 95.06 |  |
|  | Range change | -56.79 | -61.73 | -62.96 | -95.06 |  |
|  |  |  |  |  |  |  |
| *Leuciscus vorax* | Expansion | 9.87 | 9.87 | 9.21 | 10.53 | 0.904 |
|  | Reduction | 20.39 | 28.29 | 30.92 | 36.18 |  |
|  | Range change | -10.53 | -18.42 | -21.71 | -25.66 |  |
|  |  |  |  |  |  |  |
| *Rutilus lacustris* | Expansion | 0.52 | 0 | 0 | 0 | 0.916 |
|  | Reduction | 4.17 | 4.69 | 5.21 | 11.46 |  |
|  | Range change | -3.65 | -4.69 | -5.21 | -11.46 |  |
|  |  |  |  |  |  |  |
| *Rutilus frisii* | Expansion | 2.45 | 0.61 | 0.61 | 0 | 0.911 |
|  | Reduction | 12.27 | 16.56 | 16.56 | 29.45 |  |
|  | Range change | -9.82 | -15.95 | -15.95 | -29.45 |  |
|  |  |  |  |  |  |  |
| *Scardinius erythrophthalmus* | Expansion | 0 | 0 | 0 | 0 | 0.954 |
|  | Reduction | 27.74 | 36.13 | 37.42 | 81.29 |  |
|  | Range change | -27.74 | -36.13 | -37.42 | -81.29 |  |
|  |  |  |  |  |  |  |
| *Schizocypris altidorsalis* | Expansion | 0 | 0 | 0 | 0 | 0.982 |
|  | Reduction | 100 | 100 | 100 | 100 |  |
|  | Range change | -100 | -100 | -100 | -100 |  |
|  |  |  |  |  |  |  |
| *Squalius lepidus* | Expansion | 12.62 | 13.29 | 12.96 | 13.29 | 0.886 |
|  | Reduction | 11.63 | 10.96 | 10.69 | 6.31 |  |
|  | Range change | 1.00 | 2.33 | 1.99 | 6.980. |  |
|  |  |  |  |  |  |  |
| *Squalius turcicus* | Expansion | 1.27 | 0.63 | 0.32 | 0 | 0.851 |
|  | Reduction | 8.23 | 12.34 | 13.29 | 48.10 |  |
|  | Range change | -6.96 | -11.71 | -12.97 | -48.10 |  |
| *Vimba persa* | Expansion | 0 | 0 | 0 | 0 | 0.92 |
|  | Reduction | 5.41 | 8.11 | 7.43 | 12.84 |  |
|  | Range change | -5.41 | -8.11 | -7.43 | -12.84 |  |
| **Danionidae** |  |  |  |  |  |  |
| *Barilius mesopotamicus* | Expansion | 16.98 | 23.46 | 24.38 | 58.33 | 0.898 |
|  | Reduction | 26.85 | 25.31 | 25.31 | 22.22 |  |
|  | Range change | -9.88 | -1.85 | -0.93 | 36.11 |  |
|  |  |  |  |  |  |  |
| *Cabdio morar* | Expansion | 50 | 50 | 50 | 55.56 | 0.976 |
|  | Reduction | 0 | 0 | 0 | 0 |  |
|  | Range change | 50 | 50 | 50 | 55.56 |  |
| **Cyprinidae** |  |  |  |  |  |  |
| *Arabibarbus grypus* | Expansion | 2.12 | 3.33 | 6.06 | 14.85 | 0.826 |
|  | Reduction | 31.21 | 33.03 | 31.82 | 39.09 |  |
|  | Range change | -29.09 | -29.70 | -25.76 | -24.24 |  |
|  |  |  |  |  |  |  |
| *Barbus cyri* | Expansion | 0 | 0 | 0 | 0 | 0.886 |
|  | Reduction | 75.00 | 81.82 | 85.91 | 96.82 |  |
|  | Range change | -75.00 | -81.82 | -85.91 | -96.82 |  |
|  |  |  |  |  |  |  |
| *Barbus lacerta* | Expansion | 0 | 0 | 0 | 0 | 0.798 |
|  | Reduction | 4.65 | 7.56 | 7.56 | 15.12 |  |
|  | Range change | -4.65 | -7.56 | -7.56 | -15.12 |  |
|  |  |  |  |  |  |  |
| *Capoeta aculeata** | Expansion | 18.90 | 18.90 | 18.29 | 9.15 | 0.828 |
|  | Reduction | 8.54 | 14.02 | 15.24 | 42.68 |  |
|  | Range change | 10.37 | 4.88 | 3.05 | -33.54 |  |
|  |  |  |  |  |  |  |
| *Capoeta buhsei** | Expansion | 5.32 | 5.32 | 4.26 | 1.06 | 0.945 |
|  | Reduction | 3.19 | 4.26 | 5.32 | 10.64 |  |
|  | Range change | 2.13 | 1.06 | -1.06 | -9.57 |  |
|  |  |  |  |  |  |  |
| *Capoeta capoeta* | Expansion | 0 | 0 | 0 | 0 | 0.931 |
|  | Reduction | 87.88 | 96.21 | 97.73 | 100 |  |
|  | Range change | -87.88 | -96.21 | -97.73 | -100 |  |
|  |  |  |  |  |  |  |
| *Capoeta damascina* | Expansion | 0 | 0 | 0 | 0 | 0.742 |
|  | Reduction | 2.40 | 4.79 | 5.39 | 53.29 |  |
|  | Range change | -2.40 | -4.79 | -5.39 | -53.29 |  |
|  |  |  |  |  |  |  |
| *Capoeta fusca* | Expansion | 3.19 | 2.13 | 2.13 | 3.19 | 0.964 |
|  | Reduction | 3.19 | 8.51 | 8.51 | 9.57 |  |
|  | Range change | 0 | -6.38 | -6.38 | -6.38 |  |
|  |  |  |  |  |  |  |
| *Capoeta mandica** | Expansion | 28.16 | 32.04 | 26.21 | 25.24 | 0.741 |
|  | Reduction | 28.16 | 14.56 | 29.13 | 29.13 |  |
|  | Range change | 0 | 17.48 | -2.91 | -3.88 |  |
|  |  |  |  |  |  |  |
| *Capoeta razii** | Expansion | 10.43 | 10.43 | 10.43 | 10.47 | 0.846 |
|  | Reduction | 15.47 | 17.27 | 17.99 | 22.02 |  |
|  | Range change | -5.04 | -6.83 | -7.55 | -11.55 |  |
|  |  |  |  |  |  |  |
| *Capoeta saadii** | Expansion | 24.91 | 27.05 | 30.60 | 32.50 | 0.892 |
|  | Reduction | 6.76 | 7.47 | 5.69 | 5.36 |  |
|  | Range change | 18.15 | 19.57 | 24.91 | 27.14 |  |
|  |  |  |  |  |  |  |
| *Capoeta trutta* | Expansion | 12.41 | 13.45 | 13.45 | 17.93 | 0.84 |
|  | Reduction | 2.41 | 1.72 | 1.72 | 0.34 |  |
|  | Range change | 10 | 11.72 | 11.72 | 17.59 |  |
|  |  |  |  |  |  |  |
| *Capoeta umbla* | Expansion | 7.46 | 1.49 | 1.49 | 0 | 0.972 |
|  | Reduction | 10.45 | 17.91 | 14.93 | 40.30 |  |
|  | Range change | -2.99 | -16.42 | -13.43 | -40.30 |  |
|  |  |  |  |  |  |  |
| *Carasobarbus kosswigi*^△^ | Expansion | 15.33 | 16.67 | 16.67 | 18.00 | 0.936 |
|  | Reduction | 24.67 | 24.67 | 23.33 | 12.67 |  |
|  | Range change | -9.33 | -8.00 | -6.67 | 5.33 |  |
|  |  |  |  |  |  |  |
| *Carasobarbus luteus* | Expansion | 17.21 | 23.70 | 21.75 | 51.95 | 0.798 |
|  | Reduction | 38.31 | 36.69 | 36.04 | 31.82 |  |
|  | Range change | -21.10 | -12.99 | -14.29 | 20.13 |  |
|  |  |  |  |  |  |  |
| *Carasobarbus sublimus** | Expansion | 17.05 | 19.89 | 23.86 | 108.52 | 0.968 |
|  | Reduction | 0 | 0 | 0 | 0 |  |
|  | Range change | 17.05 | 19.89 | 23.86 | 108.52 |  |
|  |  |  |  |  |  |  |
| *Carassius auratus*^🞎^ | Expansion | 14.24 | 13.27 | 12.94 | 9.06 | 0.759 |
|  | Reduction | 10.03 | 15.53 | 11.33 | 21.68 |  |
|  | Range change | 4.21 | -2.27 | 1.62 | -12.62 |  |
|  |  |  |  |  |  |  |
| *Carassius gibelio*^🞎^ | Expansion | 1.38 | 0.69 | 0.69 | 0 | 0.844 |
|  | Reduction | 48.28 | 61.38 | 62.76 | 93.10 |  |
|  | Range change | -46.90 | -60.69 | -62.07 | -93.10 |  |
|  |  |  |  |  |  |  |
| *Cyprinion kais* | Expansion | 15.45 | 15.45 | 15.88 | 16.74 | 0.827 |
|  | Reduction | 15.02 | 12.02 | 10.73 | 4.29 |  |
|  | Range change | 0.43 | 3.43 | 5.15 | 12.45 |  |
|  |  |  |  |  |  |  |
| *Cyprinion macrostomus* | Expansion | 18.18 | 20.25 | 21.90 | 24.79 | 0.795 |
|  | Reduction | 0 | 0 | 0 | 0 |  |
|  | Range change | 18.18 | 20.25 | 21.90 | 24.79 |  |
| *Cyprinion tenuiradius** | Expansion | 22.14 | 24.43 | 22.14 | 22.14 | 0.945 |
|  | Reduction | 17.56 | 12.98 | 18.32 | 18.32 |  |
|  | Range change | 4.58 | 11.45 | 3.82 | 3.82 |  |
|  |  |  |  |  |  |  |
| *Cyprinus carpio*^△🞎^ | Expansion | 1.45 | 0.72 | 1.45 | 0.72 | 0.819 |
|  | Reduction | 6.52 | 7.25 | 7.97 | 11.59 |  |
|  | Range change | -5.07 | -6.52 | -6.52 | -10.87 |  |
|  |  |  |  |  |  |  |
| *Garra persica** | Expansion | 100.75 | 113.43 | 118.66 | 150.75 | 0.933 |
|  | Reduction | 0 | 0 | 0 | 0 |  |
|  | Range change | 100.75 | 113.43 | 118.66 | 150.75 |  |
|  |  |  |  |  |  |  |
| *Garra rossica* | Expansion | 49.11 | 50 | 47.32 | 43.75 | 0.95 |
|  | Reduction | 31.25 | 32.46 | 33.04 | 30.36 |  |
|  | Range change | 17.86 | 17.54 | 14.29 | 13.39 |  |
|  |  |  |  |  |  |  |
| *Garra rufa* | Expansion | 15.98 | 16.21 | 16.21 | 16.44 | 0.769 |
|  | Reduction | 31.28 | 31.05 | 31.74 | 33.11 |  |
|  | Range change | -15.30 | -14.84 | -15.53 | -16.67 |  |
|  |  |  |  |  |  |  |
| *Luciobarbus barbulus* | Expansion | 4.53 | 5.07 | 3.73 | 1.60 | 0.798 |
|  | Reduction | 31.73 | 31.20 | 36.00 | 39.73 |  |
|  | Range change | -27.20 | -26.13 | -32.27 | -38.13 |  |
|  |  |  |  |  |  |  |
| *Luciobarbus brachycephalus*^△^ | Expansion | 0 | 0 | 0 | 0 | 0.935 |
|  | Reduction | 0 | 0 | 0 | 0 |  |
|  | Range change | 0 | 0 | 0 | 0 |  |
|  |  |  |  |  |  |  |
| *Luciobarbus capito*^△^ | Expansion | 5.76 | 5.76 | 5.76 | 6.10 | 0.874 |
|  | Reduction | 1.36 | 1.02 | 1.02 | 1.69 |  |
|  | Range change | 4.41 | 4.75 | 4.75 | 4.41 |  |
|  |  |  |  |  |  |  |
| *Luciobarbus esocinus*^△^ | Expansion | 0 | 0 | 0 | 0 | 0.918 |
|  | Reduction | 0 | 0 | 0 | 0 |  |
|  | Range change | 0 | 0 | 0 | 0 |  |
|  |  |  |  |  |  |  |
| *Luciobarbus mursa* | Expansion | 4.96 | 4.55 | 4.96 | 3.31 | 0.883 |
|  | Reduction | 11.16 | 13.64 | 13.22 | 18.18 |  |
|  | Range change | -6.20 | -9.09 | -8.26 | -14.88 |  |
|  |  |  |  |  |  |  |
| *Luciobarbus subquincunciatus*^△^ | Expansion | 2.55 | 2.83 | 2.83 | 2.83 | 0.893 |
|  | Reduction | 0.28 | 0.28 | 0.28 | 0 |  |
|  | Range change | 2.27 | 2.55 | 2.55 | 2.83 |  |
|  |  |  |  |  |  |  |
| *Luciobarbus xanthopterus*^△^ | Expansion | 0.92 | 0.92 | 0.92 | 0.92 | 0.941 |
|  | Reduction | 60.37 | 62.67 | 64.98 | 68.20 |  |
|  | Range change | -59.45 | -61.75 | -64.06 | -67.28 |  |
|  |  |  |  |  |  |  |
| *Mesopotamichthys sharpeyi*^△^ | Expansion | 1.05 | 0 | 0 | 0 | 0.904 |
|  | Reduction | 98.95 | 100 | 100 | 98.42 |  |
|  | Range change | -97.89 | -100 | -100 | -98.42 |  |
|  |  |  |  |  |  |  |
| *Schizothorax pelzami* | Expansion | 0 | 0 | 0 | 0 | 0.984 |
|  | Reduction | 33.33 | 42.86 | 47.62 | 80.95 |  |
|  | Range change | -33.33 | -42.86 | -47.62 | -80.95 |  |
|  |  |  |  |  |  |  |
| *Schizothorax zarudnyi* | Expansion | 0 | 0 | 0 | 0 | 0.988 |
|  | Reduction | 100 | 100 | 100 | 100 |  |
|  | Range change | -100 | -100 | -100 | -100 |  |
| **Gobionidae** |  |  |  |  |  |  |
| *Pseudorasbora parva*^🞎^ | Expansion | 20.18 | 18.07 | 17.77 | 15.06 | 0.852 |
|  | Reduction | 10.84 | 16.57 | 19.88 | 57.23 |  |
|  | Range change | 9.34 | 1.51 | -2.11 | -42.17 |  |
|  |  |  |  |  |  |  |
| *Romanogobio macropterus* | Expansion | 0 | 0 | 0 | 0 | 0.994 |
|  | Reduction | 90.91 | 100 | 100 | 100 |  |
|  | Range change | -90.91 | -100 | -100 | -100 |  |
|  |  |  |  |  |  |  |
| *Romanogobio persus** | Expansion | 18.18 | 18.18 | 22.73 | 31.82 | 0.903 |
|  | Reduction | 0 | 0 | 0 | 0 |  |
|  | Range change | 18.18 | 18.18 | 22.73 | 31.82 |  |
| **Acheilognathidae** |  |  |  |  |  |  |
| *Rhodeus caspius* | Expansion | 7.38 | 8.05 | 8.72 | 7.38 | 0.92 |
|  | Reduction | 5.37 | 7.38 | 5.37 | 7.38 |  |
|  | Range change | 2.01 | 0.67 | 3.36 | 0 |  |
| **Xenocyprididae** |  |  |  |  |  |  |
| *Ctenopharyngodon idella*^🞎^ | Expansion | 31.51 | 33.56 | 32.88 | 41.78 | 0.827 |
|  | Reduction | 16.44 | 16.44 | 15.75 | 8.22 |  |
|  | Range change | 15.07 | 17.12 | 17.12 | 33.56 |  |
|  |  |  |  |  |  |  |
| *Hemiculter leucisculus*^🞎^ | Expansion | 6.52 | 6.52 | 6.52 | 8.70 | 0.923 |
|  | Reduction | 0.43 | 0.43 | 0.43 | 0.43 |  |
|  | Range change | 6.09 | 6.09 | 6.09 | 8.26 |  |
|  |  |  |  |  |  |  |
| *Hypophthalmichthys molitrix*^△🞎^ | Expansion | 10.44 | 10.77 | 11.11 | 13.13 | 0.841 |
|  | Reduction | 8.42 | 12.46 | 9.43 | 3.37 |  |
|  | Range change | 2.02 | -1.68 | 1.68 | 9.76 |  |
| **Tincidae** |  |  |  |  |  |  |
| *Tinca tinca* | Expansion | 0 | 0 | 0 | 0 | 0.924 |
|  | Reduction | 5.52 | 6.75 | 7.36 | 16.05 |  |
|  | Range change | -5.52 | -6.75 | -7.36 | -16.05 |  |
| **Cobitidae** |  |  |  |  |  |  |
| *Cobitis linea** | Expansion | 0 | 3.70 | 0 | 0 | 0.986 |
|  | Reduction | 92.59 | 85.19 | 92.59 | 92.59 |  |
|  | Range change | -92.59 | -81.48 | -92.59 | -92.59 |  |
|  |  |  |  |  |  |  |
| *Sabanejewia aurata* | Expansion | 0 | 0 | 0 | 0 | 0.878 |
|  | Reduction | 26.21 | 50.69 | 57.24 | 91.03 |  |
|  | Range change | -26.21 | -50.69 | -57.24 | -91.03 |  |
|  |  |  |  |  |  |  |
| *Sabanejewia caspia* | Expansion | 0 | 0 | 0 | 0 | 0.921 |
|  | Reduction | 85.38 | 90.06 | 90.06 | 97.08 |  |
|  | Range change | -85.38 | -90.06 | -90.06 | -97.08 |  |
| **Nemacheilidae** |  |  |  |  |  |  |
| *Oxynoemacheilus bergianus* | Expansion | 0 | 0 | 0 | 0 | 0.975 |
|  | Reduction | 36.43 | 41.86 | 42.64 | 93.80 |  |
|  | Range change | -36.43 | -41.86 | -42.64 | -93.80 |  |
|  |  |  |  |  |  |  |
| *Oxynoemacheilus persa** | Expansion | 5.94 | 6.93 | 5.94 | 5.94 | 0.955 |
|  | Reduction | 14.85 | 4.95 | 14.85 | 4.95 |  |
|  | Range change | -8.91 | 1.98 | -8.91 | 0.99 |  |
|  |  |  |  |  |  |  |
| *Paracobitis malapterura** | Expansion | 28.57 | 28.57 | 28.57 | 28.57 | 0.962 |
|  | Reduction | 0 | 0 | 0 | 0 |  |
|  | Range change | 28.57 | 28.57 | 28.57 | 28.57 |  |
|  |  |  |  |  |  |  |
|  |  |  |  |  |  |  |
|  |  |  |  |  |  |  |
|  |  |  |  |  |  |  |
|  |  |  |  |  |  |  |
| *Paracobitis vignai* | Expansion | 0 | 0 | 0 | 0 | 0.975 |
|  | Reduction | 100 | 100 | 100 | 100 |  |
|  | Range change | -100 | -100 | -100 | -100 |  |
|  |  |  |  |  |  |  |
| *Paraschistura bampurensis* | Expansion | 15.09 | 16.04 | 13.21 | 12.26 | 0.955 |
|  | Reduction | 16.98 | 16.98 | 16.98 | 16.98 |  |
|  | Range change | -1.89 | -0.94 | -3.77 | -4.72 |  |
|  |  |  |  |  |  |  |
| *Paraschistura cristata* | Expansion | 1.69 | 1.69 | 1.69 | 10.17 | 0.996 |
|  | Reduction | 25.42 | 23.73 | 23.73 | 8.47 |  |
|  | Range change | -23.73 | -22.03 | -22.03 | 1.69 |  |
|  |  |  |  |  |  |  |
| *Paraschistura nielseni** | Expansion | 149.12 | 185.96 | 184.21 | 201.75 | 0.804 |
|  | Reduction | 0 | 0 | 0 | 0 |  |
|  | Range change | 149.12 | 185.96 | 184.21 | 201.75 |  |
|  |  |  |  |  |  |  |
| *Sasanidus kermanshahensis** | Expansion | 0.90 | 0.90 | 0.90 | 0 | 0.942 |
|  | Reduction | 70.27 | 78.38 | 81.08 | 97.30 |  |
|  | Range change | -69.37 | -77.48 | -81.08 | -97.30 |  |
|  |  |  |  |  |  |  |
| *Turcinoemacheilus kosswigi* | Expansion | 0 | 0 | 0 | 0 | 0.876 |
|  | Reduction | 12.03 | 16.60 | 17.84 | 22.41 |  |
|  | Range change | -12.03 | -16.60 | -17.84 | -22.71 |  |
| **Bagridae** |  |  |  |  |  |  |
| *Mystus pelusius* | Expansion | 6.10 | 8.54 | 9.35 | 10.16 | 0.883 |
|  | Reduction | 50 | 58.13 | 60.16 | 70.33 |  |
|  | Range change | -43.90 | -49.59 | -50.81 | -60.16 |  |
| **Siluridae** |  |  |  |  |  |  |
| *Silurus glanis* | Expansion | 16.18 | 14.71 | 13.24 | 2.21 | 0.899 |
|  | Reduction | 29.41 | 32.35 | 33.09 | 58.09 |  |
|  | Range change | -13.24 | -17.65 | -19.85 | -55.88 |  |
|  |  |  |  |  |  |  |
| *Silurus triostegus* | Expansion | 1.79 | 1.79 | 1.79 | 1.79 | 0.944 |
|  | Reduction | 0 | 0 | 0 | 0 |  |
|  | Range change | 1.79 | 1.79 | 1.79 | 1.79 |  |
| **Sisoridae** |  |  |  |  |  |  |
| *Glyptothorax kurdistanicus* | Expansion | 5.96 | 6.38 | 6.38 | 8.51 | 0.867 |
|  | Reduction | 8.09 | 8.09 | 8.09 | 4.68 |  |
|  | Range change | -2.13 | -1.70 | -1.70 | 3.83 |  |
|  |  |  |  |  |  |  |
| *Glyptothorax silviae** | Expansion | 9.04 | 11.14 | 9.04 | 14.16 | 0.872 |
|  | Reduction | 21.08 | 17.77 | 20.18 | 14.46 |  |
|  | Range change | -12.05 | -6.63 | -11.14 | -0.30 |  |
| **Heteropneustidae** |  |  |  |  |  |  |
| *Heteropneustes fossilis*^🞎^ | Expansion | 4.63 | 4.63 | 4.63 | 5.56 | 0.939 |
|  | Reduction | 0.93 | 0.93 | 0.93 | 0.93 |  |
|  | Range change | 3.70 | 3.70 | 3.70 | 4.63 |  |
| **Salmonidae** |  |  |  |  |  |  |
| *Salmo caspius* | Expansion | 0 | 0 | 0 | 0 | 0.925 |
|  | Reduction | 100 | 100 | 100 | 100 |  |
|  | Range change | -100 | -100 | -100 | -100 |  |
|  |  |  |  |  |  |  |
| *Salmo trutta* | Expansion | 0 | 0 | 0 | 0 | 0.933 |
|  | Reduction | 65.28 | 76.85 | 77.31 | 97.69 |  |
|  | Range change | -65.28 | -76.85 | -77.31 | -97.69 |  |
| **Esocidae** |  |  |  |  |  |  |
| *Esox lucius* | Expansion | 0.41 | 0.41 | 0.41 | 0 | 0.915 |
|  | Reduction | 12.60 | 15.04 | 15.04 | 26.42 |  |
|  | Range change | -12.20 | -14.63 | -14.63 | -26.42 |  |
| **Gobiidae** |  |  |  |  |  |  |
| *Benthophilus ctenolepidus* | Expansion | 12.12 | 12.12 | 12.12 | 12.12 | 0.991 |
|  | Reduction | 24.24 | 24.24 | 24.24 | 24.24 |  |
|  | Range change | -12.12 | -12.12 | -12.12 | -12.12 |  |
| *Boleophthalmus dussumieri* | Expansion | 36.76 | 58.82 | 35.29 | 36.76 | 0.955 |
|  | Reduction | 26.47 | 22.06 | 26.47 | 26.47 |  |
|  | Range change | 10.29 | 36.76 | 8.82 | 10.29 |  |
|  |  |  |  |  |  |  |
| *Glossogobius giuris* | Expansion | 322.81 | 343.86 | 350.88 | 394.74 | 0.984 |
|  | Reduction | 0 | 0 | 0 | 0 |  |
|  | Range change | 322.81 | 343.86 | 350.88 | 394.74 |  |
|  |  |  |  |  |  |  |
| *Knipowitschia caucasica* | Expansion | 3.73 | 3.73 | 3.73 | 4.48 | 0.973 |
|  | Reduction | 0 | 0 | 0 | 0 |  |
|  | Range change | 3.73 | 3.73 | 3.73 | 4.48 |  |
|  |  |  |  |  |  |  |
| *Neogobius caspius* | Expansion | 0 | 0 | 0 | 0 | 0.97 |
|  | Reduction | 12.50 | 14.77 | 14.77 | 23.86 |  |
|  | Range change | -12.50 | -14.77 | -14.77 | -23.86 |  |
|  |  |  |  |  |  |  |
| *Neogobius melanostomus* | Expansion | 0 | 0 | 0 | 0 | 0.93 |
|  | Reduction | 85.29 | 91.91 | 92.65 | 98.53 |  |
|  | Range change | -85.29 | -91.91 | -92.65 | -98.53 |  |
|  |  |  |  |  |  |  |
| *Neogobius pallasi* | Expansion | 24.69 | 28.40 | 29.63 | 39.09 | 0.91 |
|  | Reduction | 2.06 | 3.70 | 5.35 | 3.29 |  |
|  | Range change | 22.63 | 24.69 | 24.28 | 35.80 |  |
|  |  |  |  |  |  |  |
| *Periophthalmus waltoni* | Expansion | 47.12 | 57.69 | 43.27 | 45.19 | 0.976 |
|  | Reduction | 0.96 | 0 | 0 | 9.62 |  |
|  | Range change | 46.15 | 57.69 | 43.27 | 35.58 |  |
|  |  |  |  |  |  |  |
| *Ponticola cyrius* | Expansion | 0 | 0 | 0 | 0 | 0.985 |
|  | Reduction | 97.73 | 97.73 | 97.73 | 100 |  |
|  | Range change | -97.73 | -97.73 | -97.73 | -100 |  |
|  |  |  |  |  |  |  |
| *Ponticola gorlap* | Expansion | 0 | 0 | 0 | 0 | 0.93 |
|  | Reduction | 99.11 | 100 | 100 | 100 |  |
|  | Range change | -99.11 | -100 | -100 | -100 |  |
|  |  |  |  |  |  |  |
| *Ponticola syrman* | Expansion | 0 | 0 | 0 | 0 | 0.988 |
|  | Reduction | 0 | 0 | 0 | 0 |  |
|  | Range change | 0 | 0 | 0 | 0 |  |
|  |  |  |  |  |  |  |
| *Proterorhinus nasalis* | Expansion | 0 | 0 | 0 | 0 | 0.912 |
|  | Reduction | 15.74 | 22.22 | 23.61 | 35.19 |  |
|  | Range change | -15.74 | -22.22 | -23.610 | -35.19 |  |
| **Mugilidae** |  |  |  |  |  |  |
| *Chelon saliens*^🞎^ | Expansion | 0 | 0 | 0 | 0 | 0.967 |
|  | Reduction | 1.09 | 1.09 | 1.09 | 1.09 |  |
|  | Range change | -1.09 | -1.09 | -1.09 | -1.09 |  |
|  |  |  |  |  |  |  |
| *Mugil cephalus* | Expansion | 87.50 | 233.33 | 81.25 | 81.87 | 0.984 |
|  | Reduction | 68.75 | 83.33 | 43.75 | 87.50 |  |
|  | Range change | 18.75 | 150 | 37.50 | -6.25 |  |
| **Cichlidae** |  |  |  |  |  |  |
| *Iranocichla hormuzensis** | Expansion | 10.17 | 16.95 | 20.34 | 45.76 | 0.95 |
|  | Reduction | 0 | 0 | 0 | 0 |  |
|  | Range change | 10.17 | 16.95 | 20.34 | 45.76 |  |
| **Atherinidae** |  |  |  |  |  |  |
| *Atherina caspia* | Expansion | 7.02 | 3.51 | 3.51 | 0 | 0.962 |
|  | Reduction | 17.54 | 19.30 | 17.54 | 45.61 |  |
|  | Range change | -10.53 | -15.79 | -14.04 | -45.61 |  |
| **Cyprinodontidae** |  |  |  |  |  |  |
| *Aphanius farsicus** | Expansion | 0 | 0 | 0 | 0 | 0.99 |
|  | Reduction | 100 | 100 | 100 | 100 |  |
|  | Range change | -100 | -100 | -100 | -100 |  |
|  |  |  |  |  |  |  |
| *Aphanius isfahanensis** | Expansion | 6.67 | 6.67 | 0 | 6.67 | 0.994 |
|  | Reduction | 0 | 0 | 0 | 0 |  |
|  | Range change | 6.67 | 6.67 | 0 | 6.67 |  |
|  |  |  |  |  |  |  |
| *Aphanius sophiae** | Expansion | 0 | 0 | 0 | 0 | 0.976 |
|  | Reduction | 100 | 100 | 100 | 100 |  |
|  | Range change | -100 | -100 | -100 | -100 |  |
|  |  |  |  |  |  |  |
| *Aphanius stoliczkanus* | Expansion | 76.78 | 85.34 | 87.27 | 146.82 | 0.857 |
|  | Reduction | 7.49 | 7.14 | 6.74 | 6.74 |  |
|  | Range change | 69.29 | 78.20 | 80.52 | 140.07 |  |
|  |  |  |  |  |  |  |
| *Aphanius vladykovi** | Expansion | 11.54 | 11.54 | 11.54 | 19.23 | 0.987 |
|  | Reduction | 3.85 | 3.85 | 3.85 | 3.85 |  |
|  | Range change | 7.69 | 7.69 | 7.69 | 15.38 |  |
| **Poeciliidae** |  |  |  |  |  |  |
| *Gambusia holbrooki*^🞎^ | Expansion | 66.12 | 90.23 | 96.74 | 170.03 | 0.68 |
|  | Reduction | 10.42 | 8.79 | 7.82 | 1.30 |  |
|  | Range change | 55.70 | 81.43 | 88.93 | 138.73 |  |
| **Mastacembelidae** |  |  |  |  |  |  |
| *Mastacembelus mastacembelus* | Expansion | 28.26 | 31.52 | 34.51 | 47.83 | 0.82 |
|  | Reduction | 8.97 | 8.97 | 7.88 | 6.52 |  |
|  | Range change | 19.29 | 22.55 | 26.63 | 41.30 |  |
| **Syngnathidae** |  |  |  |  |  |  |
| *Syngnathus caspius* | Expansion | 0 | 0 | 0 | 0 | 0.958 |
|  | Reduction | 45.13 | 57.52 | 58.04 | 91.15 |  |
|  | Range change | -45.13 | -57.52 | -58.04 | -91.15 |  |
| **Percidae** |  |  |  |  |  |  |
| *Perca fluviatilis* | Expansion | 0 | 0 | 0 | 0 | 0.992 |
|  | Reduction | 100 | 100 | 100 | 100 |  |
|  | Range change | -100 | -100 | -100 | -100 |  |
|  |  |  |  |  |  |  |
| *Sander lucioperca* | Expansion | 18.18 | 15.15 | 12.12 | 1.01 | 0.877 |
|  | Reduction | 48.48 | 55.56 | 68.69 | 86.87 |  |
|  | Range change | -30.30 | -40.40 | -56.57 | -85.86 |  |
| **Gasterosteidae** |  |  |  |  |  |  |
| *Gasterosteus aculeatus*^🞎^ | Expansion | 0 | 0 | 0 | 0 | 0.973 |
|  | Reduction | 7.84 | 10.78 | 11.76 | 15.69 |  |
|  | Range change | -7.84 | -10.78 | -11.76 | -15.69 |  |
|  |  |  |  |  |  |  |
| *Pungitius platygaster* | Expansion | 0 | 0 | 0 | 0 | 0.991 |
|  | Reduction | 0 | 0 | 0 | 0 |  |
|  | Range change | 0 | 0 | 0 | 0 |  |
| **Sparidae** |  |  |  |  |  |  |
| *Acanthopagrus arabicus* | Expansion | 21.90 | 25.55 | 23.36 | 20.44 | 0.952 |
|  | Reduction | 0 | 0 | 1.46 | 0.73 |  |
|  | Range change | 21.90 | 25.55 | 21.90 | 19.71 |  |

# Appendix 3. Relative importance of environmental variables estimated by MaxEnt model for each species (Units are measured in percentage). Abbreviations: A-TEM (annual mean temperature), HU (the hydrologic units occupied by the target species), ELE (elevation), Max-WID (maximum width), PRE (average annual precipitation), R-TEM (the temperature difference between the coldest and hottest months of the year), SLO (river slope).

| **Species** | A-TEM | PRE | ELE | Max-WID | SLO | R-TEM | HU |
| --- | --- | --- | --- | --- | --- | --- | --- |
| *Caspiomyzon wagneri* | 0 | 7.19 | 77.87 | 2.09 | 2.35 | 0 | 10.49 |
| *Carcharhinus leucas* | 57.77 | 0 | 23.28 | 0 | 0 | 16.01 | 2.94 |
| *Acipenser gueldenstaedtii* | 0.29 | 0 | 87.50 | 0 | 11.28 | 0 | 0.94 |
| *Acipenser persicus* | 0 | 0.37 | 77.90 | 0.10 | 0.40 | 3.20 | 18.03 |
| *Acipenser stellatus* | 2.34 | 2.11 | 84.68 | 0 | 0.26 | 6.50 | 4.11 |
| *Huso huso* | 0 | 0 | 97.64 | 0 | 0 | 0 | 2.36 |
| *Anguilla anguilla* | 0 | 0 | 81.40 | 0 | 0 | 4.55 | 14.05 |
| *Tenualosa ilisha* | 39.74 | 1.15 | 56.13 | 0.19 | 1.61 | 0.56 | 0.62 |
| *Chanos chanos* | 0 | 50.83 | 48.94 | 0.04 | 0 | 0 | 0.19 |
| *Abramis brama* | 7.46 | 1.16 | 22.76 | 3.33 | 2.77 | 4.82 | 57.70 |
| *Acanthobrama marmid* | 1.27 | 1.84 | 9.44 | 0 | 4.84 | 25.96 | 56.65 |
| *Acanthobrama microlepis* | 0 | 0 | 0 | 0 | 0 | 8.48 | 91.52 |
| *Acanthobrama persidis* | 0 | 38.66 | 3.94 | 0 | 1.96 | 10.49 | 44.95 |
| *Acanthobrama urmianus* | 0 | 1.68 | 0.14 | 0 | 2.59 | 0 | 95.59 |
| *Alburnoides eichwaldii* | 58.41 | 0.66 | 8.71 | 0 | 0.93 | 2.32 | 28.97 |
| *Alburnoides namaki* | 8.64 | 3.54 | 1.20 | 1.78 | 0 | 0.09 | 84.75 |
| *Alburnoides petrubanarescui* | 93.33 | 0 | 0 | 0.35 | 0.70 | 0 | 5.61 |
| *Alburnoides qanati* | 0 | 12.31 | 35.14 | 0 | 13.52 | 0 | 39.03 |
| *Alburnus chalcoides* | 0.20 | 7.80 | 8.00 | 0.90 | 0.40 | 0.60 | 82.10 |
| *Alburnus doriae* | 0 | 22.20 | 40.07 | 5.64 | 13.25 | 8.57 | 10.27 |
| *Alburnus filippii* | 21.61 | 4.88 | 2.32 | 1.12 | 2.41 | 14.54 | 53.13 |
| *Alburnus hohenackeri* | 1.24 | 1.43 | 6.83 | 0.79 | 3.85 | 0 | 85.86 |
| *Alburnus sellal* | 4.09 | 2.98 | 31.70 | 1.64 | 4.52 | 11.78 | 43.28 |
| *Alburnus ulanus* | 0 | 0.21 | 0 | 0 | 0 | 0 | 99.79 |
| *Arabibarbus grypus* | 66.03 | 18.15 | 0.45 | 0.34 | 1.23 | 11.24 | 2.56 |
| *Barbus cyri* | 32.49 | 3.75 | 12.55 | 1.93 | 3.98 | 2.46 | 42.84 |
| *Barbus lacerta* | 30.99 | 0 | 0.40 | 1.59 | 0.18 | 0 | 66.83 |
| *Barilius mesopotamicus* | 42.70 | 8.84 | 1.40 | 3.64 | 0 | 40.51 | 2.91 |
| *Blicca bjoerkna* | 31.81 | 0.20 | 50.92 | 0 | 17.06 | 0 | 0 |
| *Cabdio morar* | 0.27 | 79.00 | 0 | 0.09 | 0.82 | 0.25 | 19.57 |
| *Capoeta aculeata* | 14.82 | 0.74 | 3.48 | 0.68 | 0.15 | 8.80 | 71.33 |
| *Capoeta buhsei* | 3.82 | 0 | 2.33 | 0.69 | 6.69 | 0.79 | 85.68 |
| *Capoeta capoeta* | 66.41 | 7.63 | 15.40 | 0.06 | 0.42 | 0.44 | 9.64 |
| *Capoeta damascina* | 28.19 | 5.07 | 6.83 | 1.24 | 0 | 2.48 | 56.19 |
| *Capoeta fusca* | 0 | 2.67 | 2.70 | 0.81 | 3.35 | 0 | 90.47 |
| *Capoeta mandica* | 0 | 38.01 | 6.07 | 0 | 5.08 | 0 | 50.84 |
| *Capoeta razii* | 5.29 | 0 | 8.04 | 0.03 | 0.91 | 0 | 85.72 |
| *Capoeta saadii* | 15.73 | 16.84 | 47.43 | 1.45 | 1.36 | 5.64 | 11.55 |
| *Capoeta trutta* | 4.20 | 0.74 | 1.42 | 7.56 | 2.15 | 12.72 | 71.21 |
| *Capoeta umbla* | 5.40 | 33.16 | 16.56 | 2.42 | 0 | 0 | 42.47 |
| *Carasobarbus kosswigi* | 0 | 4.07 | 23.03 | 0.46 | 0.32 | 53.50 | 18.62 |
| *Carasobarbus luteus* | 23.72 | 23.67 | 7.27 | 2.86 | 4.92 | 17.40 | 20.16 |
| *Carasobarbus sublimus* | 47.72 | 0 | 8.01 | 0 | 0.08 | 0 | 44.19 |
| *Carassius auratus* | 2.50 | 25.10 | 25.00 | 3.30 | 2.00 | 1.00 | 41.10 |
| *Carassius gibelio* | 33.10 | 21.60 | 0.90 | 10.80 | 8.10 | 1.60 | 23.90 |
| *Chondrostoma orientale* | 0 | 1.84 | 0 | 20.59 | 0 | 0 | 77.57 |
| *Chondrostoma regium* | 3.74 | 6.27 | 5.13 | 4.91 | 0.93 | 0.64 | 78.38 |
| *Ctenopharyngodon idella* | 0 | 17.10 | 50.80 | 7.10 | 4.70 | 11.00 | 9.30 |
| *Cyprinion kais* | 0 | 13.09 | 0 | 0.04 | 8.71 | 12.02 | 66.14 |
| *Cyprinion macrostomus* | 9.76 | 8.19 | 3.04 | 0.02 | 1.06 | 4.39 | 73.54 |
| *Cyprinion tenuiradius* | 0 | 38.33 | 3.56 | 0.20 | 0 | 4.28 | 53.64 |
| *Cyprinus carpio* | 6.70 | 2.60 | 67.60 | 3.40 | 7.60 | 2.80 | 9.30 |
| *Garra persica* | 72.77 | 1.16 | 4.07 | 0.23 | 0.37 | 20.47 | 0.93 |
| *Garra rossica* | 1.73 | 60.64 | 13.22 | 0 | 0 | 0 | 24.41 |
| *Garra rufa* | 10.30 | 13.70 | 14.80 | 1.12 | 0 | 8.59 | 51.49 |
| *Hemiculter leucisculus* | 0 | 0 | 0 | 5.09 | 0 | 3.61 | 91.30 |
| *Hypophthalmichthys molitrix* | 0.11 | 10.76 | 42.71 | 8.51 | 0 | 7.30 | 30.61 |
| *Leuciscus aspius* | 28.61 | 3.04 | 39.65 | 4.30 | 20.37 | 1.28 | 2.75 |
| *Leuciscus vorax* | 35.55 | 2.78 | 0 | 0.41 | 5.45 | 1.24 | 54.57 |
| *Luciobarbus barbulus* | 3.69 | 10.49 | 7.34 | 3.75 | 2.17 | 8.58 | 63.99 |
| *Luciobarbus brachycephalus* | 0 | 0 | 77.82 | 2.88 | 0 | 0 | 19.30 |
| *Luciobarbus capito* | 0.70 | 3.65 | 7.66 | 5.56 | 2.52 | 7.98 | 71.93 |
| *Luciobarbus esocinus* | 0 | 4.66 | 31.01 | 0 | 1.62 | 0 | 62.71 |
| *Luciobarbus mursa* | 1.50 | 6.63 | 5.53 | 6.78 | 4.46 | 3.22 | 71.88 |
| *Luciobarbus subquincunciatus* | 0 | 0 | 15.75 | 0.19 | 0 | 15.75 | 68.32 |
| *Luciobarbus xanthopterus* | 4.48 | 0 | 3.35 | 5.52 | 25.20 | 0 | 61.45 |
| *Mesopotamichthys sharpeyi* | 45.09 | 2.99 | 38.60 | 0.86 | 2.81 | 6.53 | 3.12 |
| *Pseudorasbora parva* | 19.30 | 12.40 | 11.10 | 7.20 | 9.30 | 1.90 | 38.80 |
| *Rhodeus amarus* | 0 | 87.72 | 4.03 | 0 | 4.06 | 0 | 4.19 |
| *Romanogobio macropterus* | 41.02 | 33.99 | 9.73 | 0.75 | 2.47 | 0 | 12.04 |
| *Romanogobio persus* | 1.30 | 1.30 | 0 | 0 | 0 | 0 | 97.39 |
| *Rutilus lacustris* | 0.99 | 0 | 2.87 | 2.90 | 17.98 | 1.22 | 74.03 |
| *Rutilus frisii* | 9.34 | 24.48 | 36.43 | 0.93 | 3.69 | 11.05 | 14.08 |
| *Scardinius erythrophthalmus* | 13.49 | 0 | 49.42 | 1.72 | 35.37 | 0 | 0 |
| *Schizocypris altidorsalis* | 0 | 72.55 | 26.81 | 0 | 0 | 0.63 | 0 |
| *Schizothorax pelzami* | 0.40 | 0 | 0 | 0 | 0 | 0 | 99.60 |
| *Schizothorax zarudnyi* | 1.09 | 93.71 | 4.53 | 0 | 0 | 0.67 | 0 |
| *Squalius lepidus* | 0 | 0 | 0 | 2.45 | 0 | 1.77 | 95.79 |
| *Squalius turcicus* | 8.56 | 5.17 | 8.49 | 2.82 | 8.40 | 5.06 | 61.50 |
| *Tinca tinca* | 9.50 | 0.23 | 86.08 | 0 | 0 | 4.19 | 0 |
| *Vimba persa* | 2.53 | 2.08 | 75.45 | 2.15 | 0.18 | 0 | 17.60 |
| *Cobitis linea* | 0 | 52.97 | 0.57 | 0 | 1.06 | 0 | 45.40 |
| *Sabanejewia aurata* | 0.94 | 5.61 | 0 | 5.35 | 0 | 0 | 88.09 |
| *Sabanejewia caspia* | 64.29 | 0 | 0 | 0 | 0 | 0 | 35.71 |
| *Oxynoemacheilus bergianus* | 57.74 | 2.38 | 5.14 | 5.30 | 9.01 | 0.65 | 19.78 |
| *Oxynoemacheilus persa* | 0 | 33.70 | 10.99 | 0 | 43.62 | 0.41 | 11.28 |
| *Paracobitis malapterura* | 0 | 48.02 | 0 | 0 | 0 | 0 | 51.98 |
| *Paracobitis vignai* | 3.99 | 81.10 | 11.24 | 2.58 | 0 | 1.09 | 0 |
| *Paraschistura bampurensis* | 0 | 77.12 | 1.90 | 0.80 | 0 | 1.64 | 18.54 |
| *Paraschistura cristata* | 0 | 0 | 0 | 0 | 0 | 0.68 | 99.32 |
| *Paraschistura nielseni* | 7.23 | 20.93 | 16.54 | 0 | 4.83 | 0 | 50.47 |
| *Sasanidus kermanshahensis* | 25.31 | 1.15 | 0 | 2.74 | 1.51 | 2.27 | 67.02 |
| *Turcinoemacheilus kosswigi* | 0 | 0.57 | 7.82 | 0 | 0 | 0 | 91.62 |
| *Mystus pelusius* | 64.96 | 4.46 | 1.01 | 2.96 | 0.21 | 20.34 | 6.07 |
| *Silurus glanis* | 23.81 | 6.12 | 12.21 | 5.96 | 3.05 | 25.56 | 23.29 |
| *Silurus triostegus* | 4.02 | 0 | 29.13 | 0.56 | 28.62 | 1.71 | 35.97 |
| *Glyptothorax kurdistanicus* | 0 | 0 | 13.12 | 0 | 3.35 | 13.82 | 69.70 |
| *Glyptothorax silviae* | 12.63 | 25.46 | 11.78 | 3.66 | 0.95 | 5.37 | 40.15 |
| *Heteropneustes fossilis* | 0.60 | 0 | 41.10 | 0.40 | 2.30 | 1.30 | 54.30 |
| *Salmo caspius* | 36.17 | 4.77 | 5.49 | 3.40 | 0 | 50.17 | 0 |
| *Salmo trutta* | 50.24 | 2.01 | 2.47 | 0.23 | 11.06 | 29.16 | 4.82 |
| *Esox lucius* | 8.39 | 3.29 | 21.02 | 0.76 | 4.73 | 0 | 61.81 |
| *Benthophilus ctenolepidus* | 0 | 5.35 | 86.92 | 7.73 | 0 | 0 | 0 |
| *Boleophthalmus dussumieri* | 0 | 42.67 | 54.60 | 0.21 | 0.88 | 0.80 | 0.84 |
| *Glossogobius giuris* | 78.09 | 13.48 | 3.37 | 0 | 0 | 5.06 | 0 |
| *Knipowitschia caucasica* | 0 | 0 | 14.03 | 22.47 | 0 | 5.74 | 57.76 |
| *Neogobius caspius* | 0 | 10.46 | 88.50 | 0 | 1.04 | 0 | 0 |
| *Neogobius melanostomus* | 3.38 | 5.05 | 5.03 | 2.24 | 3.05 | 81.25 | 0 |
| *Neogobius pallasi* | 12.38 | 0.81 | 5.95 | 0.42 | 1.20 | 13.64 | 65.60 |
| *Periophthalmus waltoni* | 0 | 57.85 | 38.92 | 0.11 | 0.43 | 0 | 2.69 |
| *Ponticola cyrius* | 48.22 | 0 | 44.06 | 0.62 | 0 | 7.09 | 0 |
| *Ponticola gorlap* | 48.82 | 0.73 | 49.87 | 0 | 0.58 | 0 | 0 |
| *Ponticola syrman* | 0 | 0 | 97.04 | 2.96 | 0 | 0 | 0 |
| *Proterorhinus nasalis* | 4.74 | 0 | 0 | 2.65 | 61.66 | 2.65 | 28.30 |
| *Chelon saliens* | 0.30 | 0.40 | 57.20 | 0.60 | 0.10 | 0 | 41.40 |
| *Mugil cephalus* | 0 | 20.56 | 50.78 | 0 | 28.47 | 0 | 0.19 |
| *Iranocichla hormuzensis* | 19.35 | 0 | 5.29 | 1.26 | 0.87 | 0.82 | 72.41 |
| *Atherina caspia* | 3.20 | 0.82 | 90.67 | 5.31 | 0 | 0 | 0 |
| *Aphanius farsicus* | 0 | 59.39 | 0.38 | 0 | 0 | 0 | 40.23 |
| *Aphanius isfahanensis* | 0.65 | 3.23 | 0 | 0 | 0 | 0 | 96.12 |
| *Aphanius sophiae* | 0 | 3.06 | 0.24 | 4.04 | 0 | 0 | 92.66 |
| *Aphanius stoliczkanus* | 71.34 | 2.22 | 9.89 | 1.24 | 0.43 | 2.49 | 12.39 |
| *Aphanius vladykovi* | 0 | 1.30 | 69.20 | 8.79 | 0 | 2.25 | 18.45 |
| *Gambusia holbrooki* | 22.19 | 11.49 | 32.56 | 5.30 | 4.34 | 11.88 | 12.25 |
| *Mastacembelus mastacembelus* | 24.52 | 16.25 | 4.17 | 8.44 | 0.46 | 5.79 | 40.38 |
| *Syngnathus caspius* | 17.88 | 1.02 | 76.08 | 0.61 | 4.42 | 0 | 0 |
| *Perca fluviatilis* | 13.25 | 0.15 | 61.86 | 16.95 | 4.97 | 2.81 | 0 |
| *Sander lucioperca* | 37.52 | 5.05 | 54.16 | 0 | 1.37 | 1.74 | 0.16 |
| *Gasterosteus aculeatus* | 0.60 | 0 | 58.60 | 0.30 | 0 | 0.50 | 40 |
| *Pungitius platygaster* | 0 | 0 | 96.57 | 0 | 3.43 | 0 | 0 |
| *Acanthopagrus arabicus* | 0 | 24.54 | 46.40 | 0 | 22.67 | 0.64 | 5.75 |

# Appendix 4. New hydrologic units predicted under different scenarios for each studied species (Potential hydrologic units). *Real hydrologic units are the hydrologic units where species currently exist. Abbreviations: Urmia (Ur); Caspian (Cs); Harirood (Hr); Kavir (Kv); Tigris (Tg); Namak (Nm); Bejestan (Bj); Lut (Lu); Esfahan (Es); Kerman_Naein (Kr); Sirjan (Sr); Kor (Ko); Gulf (Gu); Jaz_Murian (Jz); Hormuz (Ho); Makran (Mk); Mashkid (Ma); Lake_Maharlu (Mh).

| **Row** | **Species** | ***Real hydrologic units** | **Potential hydrologic units in future** | | | | **Future existence in real hydrologic units** |
| --- | --- | --- | --- | --- | --- | --- | --- |
|  |  |  | **RCP 4.5 in 2050** | **RCP 4.5 in 2080** | **RCP 8.5 in 2050** | **RCP 8.5 in 2080** |  |
| 1 | *Caspiomyzon wagneri* | Cs | Cs | Cs | Cs | Cs | ✓ |
| 2 | *Carcharhinus leucas* | Tg | Tg,Gu | Tg,Gu | Tg,Gu | Tg,Gu | ✓ |
| 3 | *Acipenser gueldenstaedtii* | Cs | Cs | Cs | Cs | Cs | ✓ |
| 4 | *Acipenser persicus* | Cs | Cs | Cs | Cs | Cs | ✓ |
| 5 | *Acipenser stellatus* | Cs | Cs | Cs | Cs | Cs | ✓ |
| 6 | *Huso huso* | Cs | Cs | Cs | Cs | Cs | ✓ |
| 7 | *Anguilla anguilla* | Cs | Cs | Cs | Cs | Cs | ✓ |
| 8 | *Tenualosa ilisha* | Gu,Tg | Tg | Tg | Tg | Tg,Cs |  |
| 9 | *Chanos chanos* | Gu,Ho,Mk,Tg | Gu,Ho, Mk,Tg | Gu,Ho, Mk,Tg | Gu,Ho, Mk,Tg | Gu,Ho,Mk,Tg | ✓ |
| 10 | *Abramis brama* | Cs, Ur | Cs | Cs | Cs | Cs | ✓ |
| 11 | *Acanthobrama marmid* | Tg | Tg | Tg | Tg | Tg | ✓ |
| 12 | *Acanthobrama microlepis* | Cs | Cs | Cs | Cs | Cs | ✓ |
| 13 | *Acanthobrama persidis* | Ko, Gu, Ho | Ko, Ho, Tg, Cs, Es, Nm | Ko, Ho, Tg, Cs, Es, Nm | Ko, Ho, Tg, Cs, Es, Nm, Kr | Ko, Ho, Tg, Cs, Es, Nm, Kr | ✓ |
| 14 | *Acanthobrama urmianus* | Ur | Ur | Ur | Ur | Ur | ✓ |
| 15 | *Alburnoides eichwaldii* | Cs | Cs | Cs | Cs | 0 |  |
| 16 | *Alburnoides namaki* | Nm,Kv | Nm,Kv | Nm,Kv | Nm,Kv | Nm,Kv | ✓ |
| 17 | *Alburnoides petrubanarescui* | Ur | Ur | Ur | Ur | Ur | ✓ |
| 18 | *Alburnoides qanati* | Ko,Sr | Sr | Ko,Sr | Sr | Ko,Sr |  |
| 19 | *Alburnus chalcoides* | Cs | Cs | Cs | Cs | Cs | ✓ |
| 20 | *Alburnus doriae* | Es | Es | Es | Es | Es | ✓ |
| 21 | *Alburnus filippii* | Cs | Cs | Cs | Cs | Cs | ✓ |
| 22 | *Alburnus hohenackeri* | Cs | Cs | Cs | Cs | Cs | ✓ |
| 23 | *Alburnus sellal* | Tg,Ko, Mh, Gu, Ho | Tg,Ko, Mh, Gu, Ho | Tg,Ko, Mh, Gu, Ho | Tg,Ko, Mh, Gu, Ho | Tg,Ko, Mh, Gu, Ho | ✓ |
| 24 | *Alburnus ulanus* | Ur | Ur | Ur | Ur | Ur | ✓ |
| 25 | *Arabibarbus grypus* | Tg,Gu,Ho | Tg,Gu,Kv | Tg,Gu,Kv | Tg,Gu,  Kv | Tg,Gu, Kv,Cs |  |
| 26 | *Barbus cyri* | Cs,Ur | Cs,Ur | Cs,Ur | Cs,Ur | Cs |  |
| 27 | *Barbus lacerta* | Tg | Tg | Tg | Tg | Tg | ✓ |
| 28 | *Barilius mesopotamicus* | Tg,Gu, Ko | Tg,Gu, Ko | Tg,Gu, Ko,Nm | Tg,Gu, Ko,Nm, Kv,Es, Mh,Bj,  Ur, Cs | Tg,Gu, Ko,Nm | ✓ |
| 29 | *Blicca bjoerkna* | Cs | Cs | Cs | Cs | 0 |  |
| 30 | *Cabdio morar* | Ma,Mk | Ma,Mk | Ma,Mk | Ma,Mk | Ma,Mk | ✓ |
| 31 | *Capoeta aculeata* | Tg,Ko | Tg,Ko | Tg,Ko | Tg,Ko | Tg |  |
| 32 | *Capoeta buhsei* | Nm,Kv | Nm,Kv | Nm,Kv | Nm,Kv | Nm,Kv | ✓ |
| 33 | *Capoeta capoeta* | Cs,Ur | Cs,Ur | Cs,Ur | Cs,Ur | 0 |  |
| 34 | *Capoeta damascina* | Tg | Tg | Tg | Tg | Tg | ✓ |
| 35 | *Capoeta fusca* | Kv,Bj,  Hr, Cs | Kv,Bj,  Hr, Cs | Kv,Bj,  Hr, Cs | Kv,Bj, Hr, Cs | Kv,Bj,  Hr, Cs | ✓ |
| 36 | *Capoeta mandica* | Gu | Gu | Gu | Gu | Gu | ✓ |
| 37 | *Capoeta razii* | Cs,Tg, Nm | Cs | Cs | Cs | Cs |  |
| 38 | *Capoeta saadii* | Kr,lu,Jz,Sr,Ko, Mh,Gu,Ho,Mk | Kr,lu,Jz,Sr,Ko,Mh, Gu,Ho,Mk,Ma,Bj,Es, Kv | Kr,lu,Jz,Sr,Ko,Mh, Gu,Ho,Mk,Ma,Bj,Es,Kv,Tg | Kr,lu,Jz,Sr,Ko,Mh, Gu,Ho,Mk,Ma,Bj,Es,Kv,Tg,Nm | Kr,lu,Jz,Sr,Ko, Mh,Gu,Ho,Mk,Ma,Bj,Es,Kv,Tg, Nm,Cs |  |
| 39 | *Capoeta trutta* | Tg | Tg | Tg | Tg | Tg | ✓ |
| 40 | *Capoeta umbla* | Tg | Tg | Tg | Tg | Tg | ✓ |
| 41 | *Carasobarbus kosswigi* | Tg | Tg | Tg | Tg | Tg | ✓ |
| 42 | *Carasobarbus luteus* | Tg,Ko,Mh,Gu,Ho | Tg,Mh,Gu,Ho | Tg,Ko,Mh,Gu,Ho | Tg,Ko, Mh,Gu,Ho | Tg,Ko, Mh,Gu,Ho |  |
| 43 | *Carasobarbus sublimus* | Tg | Tg | Tg | Tg | Tg, Ho, Jz, Mk, Ma, Gu | ✓ |
| 44 | *Carassius auratus* | Cs,Ur, Tg,Gu,Ko,Mh,Ho,Bj | Cs,Ur, Tg,Mh,Kr,Bj, Lu | Cs,Ur,Tg,Mh,Kr,Bj,Lu | Cs,Ur,Tg,Mh,Kr,Bj,Lu | Cs,Ur,Tg,Kr,Mh,Bj,Lu |  |
| 45 | *Carassius gibelio* | Cs,Tg | Cs,Tg | Cs,Tg | Cs,Tg | Cs,Tg | ✓ |
| 46 | *Chondrostoma orientale* | Ko | Ko | Ko | Ko | Ko | ✓ |
| 47 | *Chondrostoma regium* | Tg,Es | Tg,Es | Tg,Es | Tg,Es | Tg,Es | ✓ |
| 48 | *Ctenopharyngodon idella* | Cs,Ur, Tg,Gu,Ko | Cs,Ur, Tg,Ko | Cs,Ur,Tg,Ko | Cs,Ur,Tg,Ko | Cs,Ur,Tg,Ko |  |
| 49 | *Cyprinion kais* | Tg,Gu | Tg | Tg | Tg | Tg |  |
| 50 | *Cyprinion macrostomus* | Tg | Tg | Tg | Tg | Tg | ✓ |
| 51 | *Cyprinion tenuiradius* | Gu | Gu | Gu | Gu | Gu | ✓ |
| 52 | *Cyprinus carpio* | Cs | Cs | Cs | Cs | Cs | ✓ |
| 53 | *Garra persica* | Ho, Mk, Jz | Ho, Mk, Jz, Ma, Gu | Ho, Mk, Jz, Ma, Gu | Ho, Mk, Jz, Ma, Gu | Ho, Mk, Jz, Ma, Gu, Mh | ✓ |
| 54 | *Garra rossica* | Mk,Jz,Ma,Lu,Bj,Kv, Hr | Mk,Jz,Ma,Lu,Bj,Kv,Hr,Es,Kr | Mk,Jz,Ma,Lu,Bj,Kv,Hr,Es,Kr | Mk,Jz, Ma,Lu, Bj,Kv,Hr,Es,Kr | Mk,Jz, Ma,Lu, Bj,Kv,Hr | ✓ |
| 55 | *Garra rufa* | Tg,Ko,Mh,Gu | Tg,Mh,Gu | Tg,Mh,Gu | Tg,Mh, Gu | Tg,Mh, Gu | ✓ |
| 56 | *Hemiculter leucisculus* | Cs | Cs | Cs | Cs | Cs | ✓ |
| 57 | *Hypophthalmichthys molitrix* | Cs,Ur, Tg,Ko | Cs,Ur, Tg,Ko,Gu | Cs,Ur,Tg,Ko,Gu | Cs,Ur,Tg,Ko,Gu | Cs,Ur,Tg,Ko,Gu | ✓ |
| 58 | *Leuciscus aspius* | Cs | Cs | Cs | Cs | Cs | ✓ |
| 59 | *Leuciscus vorax* | Tg | Tg | Tg | Tg | Tg | ✓ |
| 60 | *Luciobarbus barbulus* | Tg,Gu | Tg,Gu | Tg,Gu | Tg,Gu | Tg,Gu | ✓ |
| 61 | *Luciobarbus brachycephalus* | Cs | Cs,Ho | Cs,Ho | Cs,Ho | Cs,Ho | ✓ |
| 62 | *Luciobarbus capito* | Cs | Cs | Cs | Cs | Cs | ✓ |
| 63 | *Luciobarbus esocinus* | Tg | Tg | Tg | Tg | Tg | ✓ |
| 64 | *Luciobarbus mursa* | Cs,Ur | Cs | Cs | Cs | Cs | ✓ |
| 65 | *Luciobarbus subquincunciatus* | Tg | Tg | Tg | Tg | Tg | ✓ |
| 66 | *Luciobarbus xanthopterus* | Tg | Tg | Tg | Tg | Tg | ✓ |
| 67 | *Mesopotamichthys sharpeyi* | Tg,Gu | Tg | 0 | 0 | Tg |  |
| 68 | *Pseudorasbora parva* | Cs,Ur, Nm,Tg,Gu,Bj,Ma(all hydrologic units) | Cs,Ur,Nm,Tg,Gu,Bj,Ma,Kr,Es | Cs,Ur,Nm,Tg,Gu,Bj,Ma,Kr,Es | Cs,Ur, Nm,Tg,Gu,Bj, Es | Cs,Ur, Nm,Tg,Gu,Es |  |
| 69 | *Rhodeus amarus* | Cs,Ur, Tg | Cs,Ur | Cs,Ur | Cs,Ur | Cs,Ur |  |
| 70 | *Romanogobio macropterus* | Cs | Cs | 0 | 0 | 0 |  |
| 71 | *Romanogobio persus* | Ur | Ur | Ur | Ur | Ur | ✓ |
| 72 | *Rutilus lacustris* | Cs | Cs | Cs | Cs | Cs | ✓ |
| 73 | *Rutilus frisii* | Cs | Cs | Cs | Cs | Cs | ✓ |
| 74 | *Scardinius erythrophthalmus* | Cs | Cs | Cs | Cs | Cs | ✓ |
| 75 | *Schizocypris altidorsalis* | Bj) | 0 | 0 | 0 | 0 |  |
| 76 | *Schizothorax pelzami* | Hr,Kv | Hr,Kv | Hr,Kv | Hr,Kv | Hr |  |
| 77 | *Schizothorax zarudnyi* | Bj | 0 | 0 | 0 | 0 |  |
| 78 | *Squalius lepidus* | Tg | Tg | Tg | Tg | Tg | ✓ |
| 79 | *Squalius turcicus* | Cs, Ur | Cs, Ur | Cs, Ur | Cs, Ur | Cs, Ur | ✓ |
| 80 | *Tinca tinca* | Cs | Cs | Cs | Cs | Cs | ✓ |
| 81 | *Vimba persa* | Cs | Cs | Cs | Cs | Cs | ✓ |
| 82 | *Cobitis linea* | Ko | Ko | Ko | Ko | Ko |  |
| 83 | *Sabanejewia aurata* | Cs | Cs | Cs | Cs | Cs | ✓ |
| 84 | *Sabanejewia caspia* | Cs | Cs | Cs | Cs | Cs | ✓ |
| 85 | *Oxynoemacheilus bergianus* | Cs,Ur, Nm | Cs,Ur,Nm,Kv | Cs,Ur,Nm,Kv | Cs,Ur, Nm,Kv | Ur,Nm |  |
| 86 | *Oxynoemacheilus persa* | Ko, Gu,Mh | Ko,Mh,Cs,Kr | Ko,Mh,Cs,Kr,Nm,Es | Ko,Mh, Cs,Kr | Ko,Mh, Cs,Kr, Nm,Es |  |
| 87 | *Paracobitis malapterura* | Nm,Kv | Nm,Kv | Nm,Kv | Nm,Kv | Nm,Kv | ✓ |
| 89 | *Paracobitis vignai* | Bj | 0 | 0 | 0 | 0 |  |
| 90 | *Paraschistura bampurensis* | Mk,Jz,Ma | Mk,Jz,Ma,Lu,Ho,Bj | Mk,Jz,Ma,Lu,Ho,Bj | Mk,Jz, Ma,Lu,Ho,Bj | Mk,Jz, Ma,Lu, Ho | ✓ |
| 91 | *Paraschistura cristata* | Hr,Kv, Bj | Hr,Kv, Bj | Hr,Kv, Bj | Hr,Kv, Bj | Hr,Kv, Bj | ✓ |
| 92 | *Paraschistura nielseni* | Gu | Gu | Gu | Gu | Gu | ✓ |
| 93 | *Sasanidus kermanshahensis* | Tg | Tg | Tg | Tg | Tg | ✓ |
| 94 | *Turcinoemacheilus kosswigi* | Tg | Tg | Tg | Tg | Tg | ✓ |
| 95 | *Mystus pelusius* | Tg,Ho,Gu | Tg,Ho | Tg,Ho,Gu | Tg,Ho, Gu | Tg,Ho, Gu |  |
| 96 | *Silurus glanis* | Cs,Ur | Cs,Ur | Cs,Ur | Cs,Ur | Cs,Ur | ✓ |
| 97 | *Silurus triostegus* | Tg | Tg | Tg | Tg | Tg | ✓ |
| 98 | *Glyptothorax kurdistanicus* | Tg | Tg | Tg | Tg | Tg | ✓ |
| 99 | *Glyptothorax silviae* | Tg, Gu | Tg, Gu | Tg, Gu | Tg, Gu | Tg, Gu | ✓ |
| 100 | *Heteropneustes fossilis* | Tg | Tg | Tg | Tg | Tg | ✓ |
| 101 | *Salmo caspius* | Cs | 0 | 0 | 0 | 0 |  |
| 102 | *Salmo trutta* | Cs,Ur, Nm | Cs,Ur | Cs,Ur | Cs,Ur | Cs |  |
| 103 | *Esox lucius* | Cs | Cs | Cs | Cs | Cs | ✓ |
| 104 | *Benthophilus ctenolepidus* | Cs | Cs | Cs | Cs | Cs | ✓ |
| 105 | *Boleophthalmus dussumieri* | Tg,Gu,Ho,Mk | Gu,Ho,Mk | Tg,Gu,Ho,Mk | Gu,Ho,Mk | Gu,Ho,Mk |  |
| 106 | *Glossogobius giuris* | Ho,Mk | Tg,Gu,Ho,Mk,Jz,Lu | Tg,Gu,Ho,Mk,Jz,Lu | Tg,Gu, Ho,Mk, Jz,Lu | Tg,Gu, Ho,Mk, Jz,Lu,Bj | ✓ |
| 107 | *Knipowitschia caucasica* | Cs | Cs | Cs | Cs | Cs | ✓ |
| 108 | *Neogobius caspius* | Cs | Cs | Cs | Cs | Cs | ✓ |
| 109 | *Neogobius melanostomus* | Cs | Cs | Cs | Cs | Cs | ✓ |
| 110 | *Neogobius pallasi* | Cs | Cs | Cs | Cs | Cs | ✓ |
| 111 | *Periophthalmus waltoni* | Tg,Gu,Ho,Mk | Tg,Gu,Ho,Mk | Tg,Gu,Ho,Mk | Tg,Gu, Ho,Mk | Tg,Gu, Ho,Mk | ✓ |
| 112 | *Ponticola cyrius* | Cs | Cs | Cs | Cs | 0 |  |
| 113 | *Ponticola gorlap* | Cs | Cs | 0 | 0 | 0 |  |
| 114 | *Ponticola syrman* | Cs | Cs | Cs | Cs | Cs | ✓ |
| 115 | *Proterorhinus nasalis* | Cs | Cs | Cs | Cs | Cs | ✓ |
| 116 | *Chelon saliens* | Cs | Cs | Cs | Cs | Cs | ✓ |
| 117 | *Mugil cephalus* | Tg,Mk | Tg,Mk,Ho | Tg,Mk,Ho,Gu | Tg,Mk, Ho | Mk,Ho |  |
| 118 | *Iranocichla hormuzensis* | Ho | Ho | Ho | Ho | Ho | ✓ |
| 119 | *Atherina caspia* | Cs | Cs | Cs | Cs | Cs | ✓ |
| 120 | *Aphanius farsicus* | Mh | 0 | 0 | 0 | 0 |  |
| 121 | *Aphanius isfahanensis* | Es | Es | Es | Es | Es | ✓ |
| 122 | *Aphanius sophiae* | Ko | 0 | 0 | 0 | 0 |  |
| 123 | *Aphanius stoliczkanus* | Tg, Ho,Gu,Mk,Ma | Tg, Ho,Gu,Mk,Ma | Tg, Ho,Gu,Mk,Ma,Jz | Tg, Ho,Gu,Mk,Ma, Jz | Tg, Ho,Gu,Mk,Ma, Jz,Bj,Kr,Sr,Es,Ko,Mh, Nm,Kv | ✓ |
| 124 | *Aphanius vladykovi* | Tg | Tg, Cs | Tg, Cs | Tg, Cs | Tg, Cs | ✓ |
| 125 | *Gambusia holbrooki* | Cs,Ur, Nm,Tg,Es,Ko,Mh,Gu,Ho,Mk, Jz,Ma, Lu,Bj | 19 hydrol-ogic units-2 hydrol-ogic units(hr,Bj) | 19 hydrologic units-2 hydrologic units(hr,Bj) | 19 hydrologic units | 19 hydrolo-gic units | ✓ |
| 126 | *Mastacembelus mastacembelus* | Tg,Gu | Tg,Gu | Tg,Gu | Tg,Gu | Tg,Gu | ✓ |
| 127 | *Syngnathus caspius* | Cs | Cs | Cs | Cs | Cs | ✓ |
| 128 | *Perca fluviatilis* | Cs | 0 | 0 | 0 | 0 |  |
| 129 | *Sander lucioperca* | Cs | Cs | Cs | Cs | Cs | ✓ |
| 130 | *Gasterosteus aculeatus* | Cs | Cs | Cs | Cs | Cs | ✓ |
| 131 | *Pungitius platygaster* | Cs | Cs | Cs | Cs | Cs | ✓ |
| 132 | *Acanthopagrus arabicus* | Gu, Tg,Ho,Mk | Gu, Tg,Ho, Mk | Gu, Tg,Ho, Mk,Cs | Gu, Tg,Ho, Mk | Gu, Tg,Ho, Mk,Cs | ✓ |

**Appendix 5.** Fish species richness in any site at current and different future scenarios per HU.

**
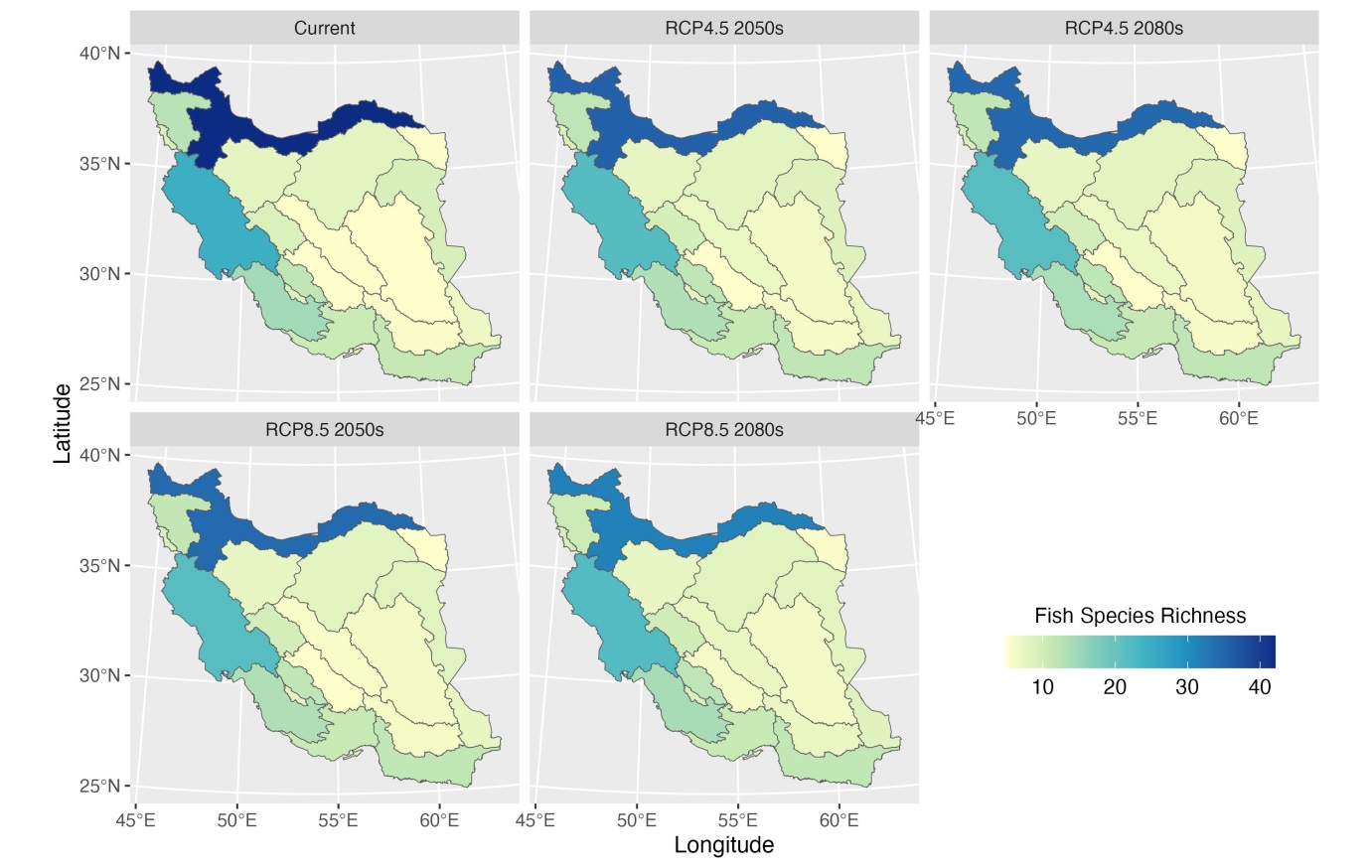
**

**Appendix 6.** Fish species richness change in different future scenarios.


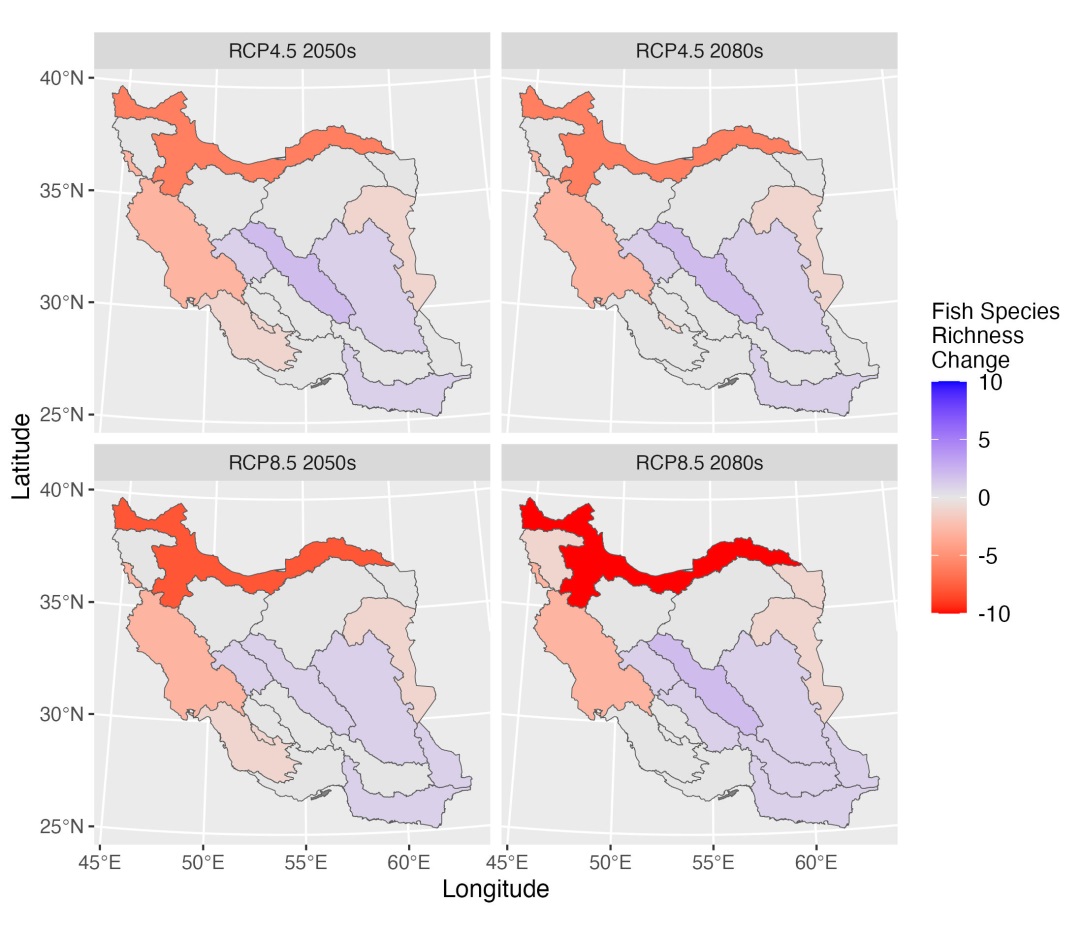

Supplement: Supplementary file 1 — Supplementary Information 1. [file 41598_2023_41406_MOESM1_ESM.docx]
